# Occurrence of Hepatitis A Virus in Water Matrices: A Systematic Review and Meta-Analysis

**DOI:** 10.3390/ijerph20021054

**Published:** 2023-01-06

**Authors:** Guy Roussel Takuissu, Sebastien Kenmoe, Jean Thierry Ebogo-Belobo, Cyprien Kengne-Ndé, Donatien Serge Mbaga, Arnol Bowo-Ngandji, Juliette Laure Ndzie Ondigui, Raoul Kenfack-Momo, Serges Tchatchouang, Josiane Kenfack-Zanguim, Robertine Lontuo Fogang, Elisabeth Zeuko’o Menkem, Ginette Irma Kame-Ngasse, Jeannette Nina Magoudjou-Pekam, Carolina Veneri, Pamela Mancini, Giusy Bonanno Ferraro, Marcello Iaconelli, Lidia Orlandi, Claudia Del Giudice, Elisabetta Suffredini, Giuseppina La Rosa

**Affiliations:** 1 Centre for Food, Food Security and Nutrition Research, Institute of Medical Research and Medicinal Plants Studies, Yaounde, Cameroon; 2 Department of Microbiology and Parasitology, University of Buea, Buea, Cameroon; 3 Medical Research Centre, Institute of Medical Research and Medicinal Plants Studies, Yaounde, Cameroon; 4 Epidemiological Surveillance, Evaluation and Research Unit, National AIDS Control Committee, Yaounde, Cameroon; 5 Department of Microbiology, The University of Yaounde I, Yaounde, Cameroon; 6 Department of Biochemistry, The University of Yaounde I, Yaounde, Cameroon; 7 Scientific Direction, Centre Pasteur du Cameroun, Yaounde, Cameroon; 8 Department of Animal Biology, University of Dschang, Dschang, Cameroon; 9 Department of Biomedical Sciences, University of Buea, Buea, Cameroon; 10 Department of Environment and Health, Istituto Superiore di Sanità, 00161 Rome, Italy; 11 Department of Food Safety, Nutrition and Veterinary Public Health, Istituto Superiore di Sanità, 00161 Rome, Italy

**Keywords:** HAV, Hepatitis A virus, water matrices, prevalence

## Abstract

Hepatitis A is a common form of viral hepatitis. It is usually transmitted through the ingestion of contaminated food and water. This systematic review was carried out to summarise the overall prevalence of Hepatitis A virus (HAV) in different water matrices: untreated and treated wastewater, surface water, groundwater, drinking water, and others (e.g., irrigation water and floodwater). The literature search was performed in four databases: PubMed, Web of Science, Global Index Medicus, and Excerpta Medica Database. Heterogeneity (I^2^) was assessed using the χ^2^ test on the Cochran Q statistic and H parameters. A total of 200 prevalence data from 144 articles were included in this meta-analysis. The overall prevalence of HAV in water matrices was 16.7% (95% CI: 13.4–20.3). The prevalence for individual matrix was as follows: 31.4% (95% CI: 23.0–40.4) untreated wastewater, 18.0% (95% CI: 9.5–28.2) treated wastewater, 15.0% (95% CI: 10.1–20.5) surface water, 2.3% (95% CI: 0.1–6.0) in groundwater, 0.3% (95% CI: 0.0–1.7) in drinking water, and 8.5% (95% CI: 3.1–15.6) in other matrices. The prevalence was higher in low-income economies (29.0%). Africa and Eastern Mediterranean were the regions with higher HAV prevalence values. This study showed a high heterogeneity (I^2^ > 75%) with a significant publication bias (*p* value Egger test < 0.001). The results of this review suggest that water matrices could be an important route of HAV transmission even in industrialized countries, despite the lower prevalence compared to less industrialized countries, and the availability of advanced water management systems. More effective water/wastewater treatment strategies are needed in developing countries to limit the environmental circulation of HAV.

## 1. Introduction

Hepatitis A is a highly contagious liver infection [1], first discovered in 1973 [2]. It can cause mild to severe illness and occasionally acute liver failure, which is often fatal. Unlike hepatitis B and C, hepatitis A does not cause chronic liver disorders. In a minority of cases, it can lead to extrahepatic manifestations, including urticarial and maculopapular rash, acute kidney injury, autoimmune hemolytic anemia, aplastic anemia, acute pancreatitis, glomerulonephritis, and thrombocytopenia [3,4]. Approximately 1.5 million people are infected with hepatitis A each year; however, this figure seems to be underestimated, and the infection rate is probably much higher [5], making hepatitis A a global public health concern. According to the World Health Organization (WHO), there were 7134 deaths worldwide caused by this disease in 2016, accounting for 0.5% of the mortality due to viral hepatitis [6]. The disease is caused by hepatitis A virus (HAV), an RNA virus of the *Picornaviridae* family, genus Hepatovirus. Six genotypes of HAV are currently recognized (genotypes I–VI). Genotypes I, II, and III, each divided into subtypes A and B, infect humans [7,8,9]. HAV is mainly transmitted via the faecal–oral route through contaminated food or water. Drinking water can be a route of major concern for HAV outbreaks; moreover, water-borne outbreaks have resulted from contaminated water supplied in households due to damages or locations of water pipelines close to drain or sewerage systems [10]. Endemicity is high in low-income countries, mainly due to inadequate safe water and poor sanitation and hygiene [11,12]. In these countries, most children (90%) are infected with HAV before the age of 10 years, often without symptoms [6]. Conversely, infection rates are low in high-income countries with good sanitary and hygienic conditions. Limiting environmental contamination is essential to control hepatitis A, hence the importance of understanding environmental sources of HAV. Several studies have reported the detection of HAV in foods, untreated and treated wastewater, and other water environments [13]. 

Several systematic reviews on HAV in water or food have been published, focusing on certain foods or specific water matrices. Chatziprodromidou et al., showed that HAV was the second most abundant virus in fresh produce [14]. Bellou et al., found that HAV was one of the most common viral pathogens detected in shellfish [15]. Thébault et al. demonstrated the critical role of untreated drinking water, shellfish, and crop products in sporadic hepatitis A infection [16]. A recent review by Gholipour and co-workers highlighted that HAV is frequently found in sewage sludge [17]. Boehm et al. reviewed decay rates of waterborne viruses, including HAV, in surface waters [18]. Finally, Kuodi and coworkers characterised the environmental presence of HAV in low- and middle-income countries, showing that HAV occurrence declined by 10% over the review period (2005–2019) [19]. No systematic review, however, has looked comprehensively at all water matrices thus far, which is important to understand the extent of viral contamination of water environments. Therefore, the objective of this systematic review and meta-analysis was to assess the overall prevalence of HAV in different water matrices, including untreated and treated wastewater, surface water, groundwater, and drinking water.

## 2. Materials and Methods

### 2.1. Protocol and Registration

This systematic review and meta-analysis was conducted between March and November 2022. The review protocol was registered on Prospero, number CRD42021289116. This study was designed by the Preferred Reporting Items for Systematic Reviews and Meta-Analysis (PRISMA) standard guidelines [20].

### 2.2. Eligibility Criteria

We included all studies published worldwide from inception until March 2022.

### 2.3. Inclusion and Exclusion Criteria

We included studies that met the following criteria: (a) containing data about the prevalence of HAV in water matrices, (b) original studies, and (c) published in English or French. Letters to editors, comment papers, brief reports, research news, systematic review, meta-analysis, and studies with a number of tested samples (sample size) equal or below 10 were excluded.

### 2.4. Information about Searches

We searched four databases including PubMed, Excerpta Medica Database (Embase), Web of Science, and Global Index Medicus. The search terms were related to HAV and water matrices (see search strategy in Table S1). A manual search in the list of references of relevant studies was also conducted to identify any additional articles missed by the online search.

### 2.5. Data Extraction and Management

Firstly, duplicate articles were removed. Two reviewers screened the titles and abstracts of all the articles using Rayyan—Intelligent Systematic Review website (https://www.rayyan.ai/ (accessed on 8 April 2022)). In case of discrepancies, a third reviewer intervened as a referee. After the preliminary screening, the data were extracted from the selected studies using a pre-designed Google data abstraction form. The different data extracted were: name of the first author, year of publication, study period, sampling approach (probabilistic/non-probabilistic), number of sites (monocenter, multicenter and nationally representative), timing of samples collection (prospective, retrospective), country, WHO region, United Nations Statistics Division (UNSD) region [21], country income level [22], type of water matrix (untreated wastewater, treated wastewater, surface water, drinking water, groundwater, and others), sample size, detection method, detected genotypes, and viral loads. In case different prevalence data were obtained for the same samples according to the HAV concentration and/or detection methods, the estimate with the highest prevalence was included. In case water samples were collected at different stages of treatment, we calculated the sum of all the categories of water and considered a single category of treated water. After data extraction, two reviewers screened the data extracted from all included studies.

### 2.6. Quality Assessment

To assess the quality of the studies, we used the tool developed by Hoy et al. for prevalence studies; this allowed the included studies to be evaluated for risk of bias (Table S2) [23]. Items for risk of bias assessment included external validity controls (e.g., representativity at national level of the prevalence data, sampling size and frame) and internal validity controls (e.g., use of valid and reliable detection assays, acceptable water matrix definition, length of the study period >1 year, etc.).

### 2.7. Statistical Analysis

Study-specific estimates were pooled using a random-effects model meta-analysis from DerSimonian and Laird [24]. Heterogeneity was assessed by the Cochrane Q statistical test and quantified by I^2^ values, assuming that the I² values of 25%, 50%, and 75% represent low, moderate, and high heterogeneity, respectively [25]. Publication bias was assessed by Egger’s test and the funnel plot [26]. Sensitivity analysis was carried out using studies that had a low risk of bias and studies in which a process control virus (e.g., murine norovirus, mengovirus, etc.) was added to the samples before analysis, in order to monitor all the analytical steps. Subgroup analyses were conducted according to sampling approach, setting, country, WHO and UNSD regions, country income level, and water matrix. A *p*-value < 0.05 indicated a significant difference. R software version 4.1.0 was used to perform analyses [27,28].

## 3. Results

### 3.1. Study Selection

**Figure 1:**
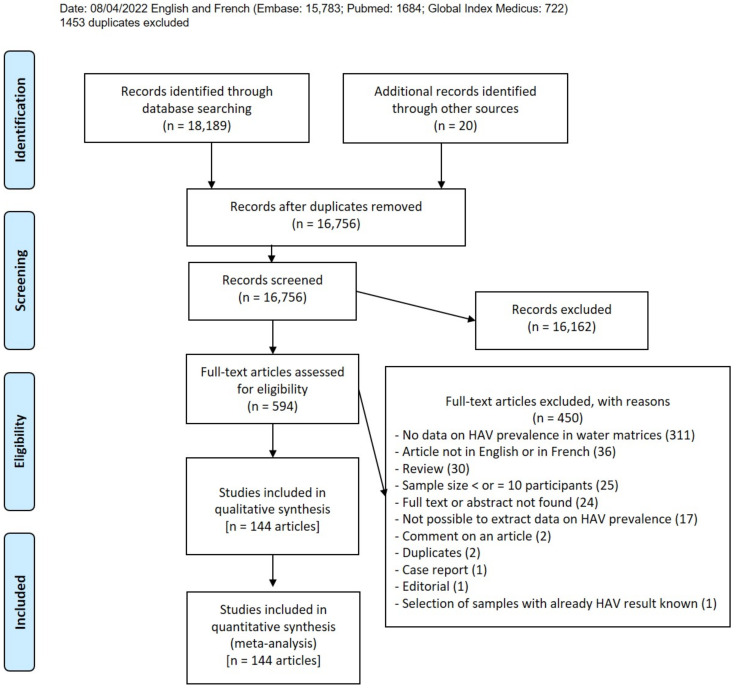
Study selection.

After searching the four databases, we obtained 18,189 articles; an additional 20 papers were included as results of manual searches (Figure 1). A total of 1453 duplicates were removed, as well as 16,162 articles for unfitting titles or abstracts (articles that do not meet inclusion criteria). A total of 594 articles were recorded as eligible, 450 of which were excluded for reasons given in Table S3. Finally, 144 articles were included in this systematic review (Table S5), which resulted in 200 HAV prevalence data from six different water matrices [29,30,31,32,33,34,35,36,37,38,39,40,41,42,43,44,45,46,47,48,49,50,51,52,53,54,55,56,57,58,59,60,61,62,63,64,65,66,67,68,69,70,71,72,73,74,75,76,77,78,79,80,81,82,83,84,85,86,87,88,89,90,91,92,93,94,95,96,97,98,99,100,101,102,103,104,105,106,107,108,109,110,111,112,113,114,115,116,117,118,119,120,121,122,123,124,125,126,127,128,129,130,131,132,133,134,135,136,137,138,139,140,141,142,143,144,145,146,147,148,149,150,151,152,153,154,155,156,157,158,159,160,161,162,163,164,165,166,167,168,169,170,171,172,173].

### 3.2. Study Characteristics

The characteristics of the included studies are listed in Tables S4–S6. The studies included were published between 1987 and 2022, and the sampling period ranged from 1986 to 2020. Most of the studies were non-probabilistic 195/200 (97.5%), prospective 197/200 (98.5%), and multicenter 130/200 (65.0%). The most represented UNSD Region was Southern Europe (53/200, 26.5%), while the most reported WHO Regions were Europe (74/200, 37.0%) and America (49/200, 24.5%). The most represented countries were Italy (29/200, 14.5%) and Brazil (22/200, 11.0%). High-income countries were prevalent (107/200, 53.5%), followed by upper-middle income countries (54/200, 27.0%). The water matrices were categorized into six groups, and the most represented were surface waters (66/200, 33.0%), followed by untreated wastewater (56/200, 28.0%) (Table S4). As for HAV genotypes, IA was the most frequently detected (749 samples), followed by IB (391 samples), IIIA (17 samples), and V (1 sample) (Table S5). Only nine studies reported information on the presence of infectious HAV by isolation in cell lines [47,66,85,88,132,145,150,158,166] (Table S5). Attempts to isolate HAV was carried out on Buffalo green monkey kidney cells (BGMK), PLC/PRF/5 human liver cells, Colorectal adenocarcinoma cells (Caco-2), Fetal rhesus monkey kidney (FRHK-4), Human lung carcinoma epithelial cells (A549), Verda reno cells (VERO), African rhesus monkey kidney cells (MA104), and Hep-2. Conventional RT-PCR (109/200, 54.5%) and real-time RT-PCR (75/200, 37.5%) were the analytical approaches most frequently used. Of the 75 studies using real-time RT-PCR, 34 obtained quantitative data, viral loads ranging from ˂LOD to 3.70 × 10^10^ genome copies (gc)/L. The concentration ranges were <1 to 3.7 × 10^10^ for untreated wastewater, 2.3 × 10^1^ to 3.3 × 10^7^ for treated wastewater, 1.5 × 10^1^ to 10^7^ for surface waters and no data for drinking water and groundwater. Moreover, low concentrations were reported in “other matrices” (6.87 × 10^−1^ to 7.4 × 10^−1^). An analytical process control to assess the performance of concentration and extraction of samples was present in 74/200 (37.0%) of the studies; the risk of bias was low in 13/200 studies (6.5%) and moderate in the remaining 187/200 (93.5%) (Table S4).

### 3.3. HAV Prevalence in Water Matrices

**Figure 2:**
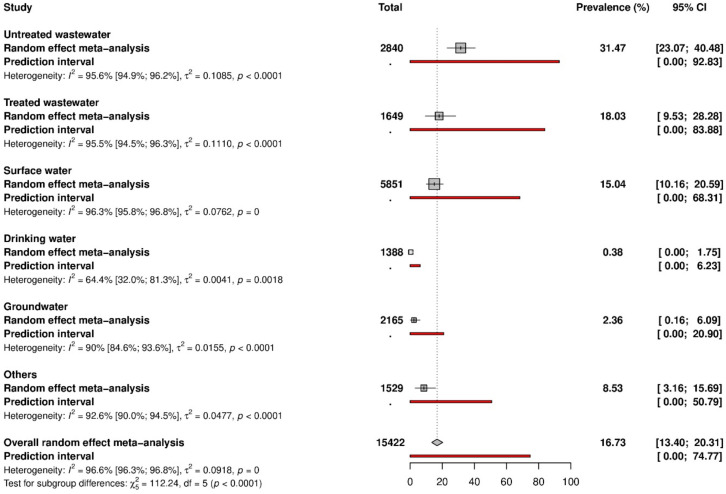
Data on HAV prevalence.

Data on HAV prevalence are represented in Figure 2 and Figure S1. The global HAV prevalence in water environments was 16.7% (95% CI: 13.4–20.3). According to the different water matrices, prevalence varied from 0.3% to 31.5%. In detail, the prevalence was as follows: 31.5% (95% CI: 23.1–40.5) in untreated wastewater, 18.0% (95% CI: 9.5–28.3) in treated wastewater, 15.0% (95% CI: 10.2–20.6) in surface water, 2.4% (95% CI: 0.2–6.1) in groundwater, 0.4% (95% CI: 0.0–1.8) in drinking water. Prevalence in other water types was 8.5% (95% CI: 3.2–15.7).

### 3.4. Heterogeneity, Publication Bias and Sensitivity Analysis

Table 1 and Figure S2 present the degree of heterogeneity and publication bias. Significant heterogeneity (H ˃ 1 and I^2^ ˃ 75%) and publication bias (*p* < 0.05 for Egger’s test) were found to be associated with the estimation of prevalence data in the different water matrices. The publication bias results obtained by Egger’s test were confirmed by the funnel plot (Figure S2). Due to the limited number of studies with low risks of bias, the results of the sensitivity analysis displayed under- (untreated wastewater) or overestimation (surface water) compared to global results (Table 1). In studies applying a process control, on the other hand, the results closely approximated the global results, pointing out the robustness of the analysis.

### 3.5. Subgroup Analyses

**Figure 3:**
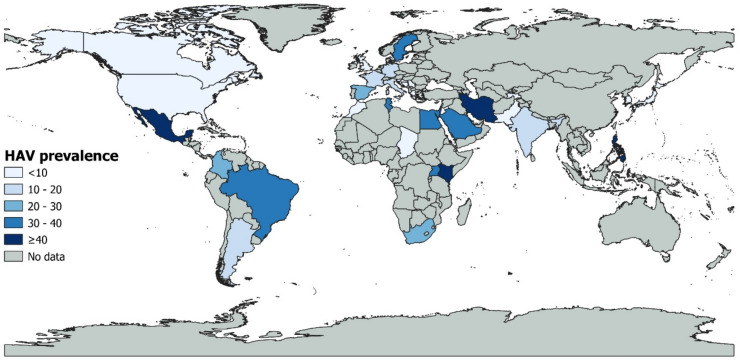
Global HAV prevalence.

Table S7 presents the subgroup analysis. The global prevalence was significantly different according to countries (*p* < 0.001) with higher prevalence in Kenya (52.0%, 95% CI: 0.0–100, 3 prevalence data), followed by Tunisia (39.1%, 95% CI: 21.8–57.8, 9 prevalence data), and Uganda (36.8%, 95% CI: 20.7–54.3, 5 prevalence data) (Figure 3). Italy and Brazil, the most represented countries, had a prevalence of 11.5% and 32.5%, respectively. According to the WHO region, a significantly higher prevalence (*p* < 0.001) was found in Africa (31.8%, 95% CI: 16.9–48.8, 22 prevalence data), followed by Eastern Mediterranean (23.7%, 95% CI: 13.4–35.6, 24 prevalence data) and America (20.0%, 95% CI: 13.2–27.9, 49 prevalence data). For UNSD region, higher prevalence (*p* < 0. 001) was in Eastern Africa (43.2%, 95% CI: 12.5–77, 8 prevalence data), followed by Northern Africa (34.4%, 95% CI: 20.7–49.4, 14 prevalence data), Southern America (30.0%, 95% CI: 17.5–44.2, 26 prevalence data), and Southern Africa (28.5%, 95% CI: 13.0–47.0, 13 prevalence data). Regarding country income level, the higher prevalence (*p* < 0.001) was in low-income economies (29.0%, 95% CI: 11.8–49.6, 6 prevalence data), while high-income economies, which are the most represented, had the prevalence of 10.8% (95% CI: 7.8–14.1, 107 prevalence data).

No statistically significant difference in the prevalence values was found according to time periods (Table S7): 14.5% during 1986–2000 (25 studies), 17.3% during 2000–2010 (67 studies), and 15.5 % during 2010–2020 (75 studies).

## 4. Discussion

Hepatitis A virus is one of the most important causes of acute viral hepatitis worldwide. The virus is present in all regions of the world, both industrialized and non-industrialized countries, making hepatitis A a significant health problem regardless of the country’s economic level. HAV is common in areas with insufficient sanitation and limited access to clean water [174]. In highly endemic areas, epidemics of hepatitis A are uncommon, and a large proportion of adults in the population are immune. Instead, in areas of intermediate endemicity, adolescents and adults are susceptible to infection, and outbreaks are more frequent. In areas of low endemicity, infection is less common, but disease occurs among people in high-risk groups (e.g., people travelling to areas of high endemicity) and as community-wide outbreaks [18]. HAV can be transmitted through ingestion of contaminated water; indeed viral contamination of water environments has been frequently reported as a primary source of hepatitis outbreaks [18]. Thus, understanding the occurrence of HAV in water environments is crucial to better understand the epidemiology of the disease. However, the few reviews published to date only addressed specific water environments [16,17,175] or a specific subset of countries [20].

Here, we aimed at summarizing all the existing literature on the prevalence of HAV in waters, including untreated and treated wastewater, surface water, groundwater, and drinking water. A total of 144 articles and 200 prevalence data were included in this study, spanning a period of 34 years (1986–2020). The overall prevalence of HAV in water was 16.7%, varying significantly according to the degree of water quality. As expected, the prevalence was higher in untreated wastewater (31.4%), with viral concentrations reaching up to 3.7 × 10^10^ gc/L. Virus occurrence in sewage samples may reflect the epidemiology pattern of virus infections in the population; it is the result of the high viral excretion by infected individuals, ranging from 10^6^ to 10^11^ viral particles per gram of faeces [176]. Despite the improvement in wastewater treatment technology, water treatments are in some cases unable to provide virus-free wastewater effluents. Indeed, a prevalence of 18.0% and a viral concentration up to 3.3 × 10^7^ cg/L was detected in treated wastewater, though this value reflects the average detection of HAV in wastewater at different levels of treatment and may therefore overestimate its occurrence in the finally treated wastewater. Viruses can be introduced to surface waters through the discharge of untreated or improperly treated sewage. Indeed, a prevalence of 15.0% and maximum concentrations up to 10^7^ cg/L was found in surface waters in this study—including rivers, lakes, marine waters—which are the water environments collecting treated wastewaters. Interestingly, the HAV prevalence in groundwater was 2.3%. Contaminated groundwater has indeed been associated with HAV outbreaks. For example, in USA, the Waterborne Disease and Outbreak Surveillance System (WBDOSS) reported HAV as the most commonly reported etiology for outbreaks associated with untreated groundwater in the period of 1971–2008, suggesting that individual water systems that used wells with untreated groundwater is a common waterborne exposure pathway for HAV [7]. Factors contributing to groundwater contamination include nearby septic systems or sewage, heavy rainfall, improper well construction and maintenance as well as surface water infiltration.

Finally, low HAV prevalence was found in drinking water (0.3%, with only 11 available studies), suggesting the effectiveness of drinking water treatment technologies. These usually include conventional pre-treatments (coagulation, sedimentation, and filtration), and disinfection (e.g., chlorination, UV) to allow pathogens’ inactivation and guarantee the safety of drinking water. However, despite the low prevalence observed in drinking waters, these can be potential sources of HAV contamination. In fact, due to the low infectious dose of HAV, the presence of the virus even at low concentrations can be enough to cause infection.

The regions with the highest HAV prevalence were Africa and Eastern Mediterranean. These regions are also strongly represented by low-income countries, confirming the study results showing that low-income economies had the highest HAV prevalence (29.0%). It has already been demonstrated that the socioeconomic status is strongly associated with HAV seroprevalence [177], and low-income economies represent endemic areas [12,13]. The endemicity of the disease in these regions is thought to be a consequence of water contamination, as these regions are also known for poor access to good quality water, poor sanitation, and lack of financial resources to set up adequate water treatment systems. Most of the European Union (EU) and European Economic Area (EEA) is considered a region of very low HAV endemicity; however, geographical differences exist, supporting the need to reconsider specific prevention and control measures, to further decrease HAV circulation [177].

The differences in prevalence observed in the studies of this review may also depend on the variety of methods used. Indeed, a wide variety of HAV detection methods were used in the collected studies; conventional RT-PCR was the most common (54.5%), followed by real-time PCR (37.5%). Data on HAV genotypes showed that IA was the most frequently detected, followed by IB; genotype IIIA was also detected in some cases. This is in agreement with epidemiological data, on the global distribution of HAV genotypes: genotype I is the most prevalent worldwide, with IA being reported more frequently than IB, and sub-genotype IIIA is prevalent in central Asia (https://www.ecdc.europa.eu/en/hepatitis-A/facts (accessed on 11 November 2022)). In areas of low endemicity, sub-genotype IA dominates, but all genotypes and subtypes have been reported [177].

Prevalence data over time (periods [1986–2000], [2000–2010], and [2010–2020]) showed no statistically significant differences or trends. Different factors may have contributed to such a result in one direction or another, such as the steady improvement of hygiene conditions worldwide, the increased sensitivity of analytical methods, and the progressive increasing number of studies from low and low/middle income economies.

Only 6.5% of the studies included in this review had a low risk of bias, which calls into question the methodological quality of the studies (e.g., errors in design, analysis, or reporting; missing information). A high level of heterogeneity of the data (I^2^ ˃ 75%) was also observed in this review, with only 3.0% of the studies conducted in low-income economies, in areas of high endemicity and low access to good quality water. This low percentage would considerably reduce the overall prevalence obtained. Publication bias was detected in association with the estimated prevalences in the different water groups (*p* < 0.05 for Egger’s test). This may point to underreporting of negative results obtained in monitoring studies, or may derive from an a priori selection of areas of investigation, prioritizing environmental studies targeting health-relevant viral pathogens with known high prevalence in the population. This may explain, for example, the unbalance of studies between northern/western and southern Europe in this review (19 studies vs. 53).

## 5. Conclusions

In conclusion, the results demonstrate that, except for drinking water where low HAV prevalence was observed, the other water matrices, and in particular untreated wastewater, can constitute a fairly important source of HAV contamination. However, based on the data, the water treatments significantly reduce the occurrence of HAV, suggesting that effective wastewater, surface water, and drinking water management systems are the key in the fight against waterborne hepatitis A.

## Figures and Tables

**Table 1:** Summary of global meta-analysis results for the prevalence of hepatitis A virus in different water matrices divided by risk of bias and by use of process control.

Water Matrix	Prevalence (%) [95%CI]	95% Prediction Interval	N° of Studies	N° of Samples	H [95%CI]	I^2^ [95%CI]	*p* Heterogeneity
**Untreated wastewater**							
Overall	31.5 [23.1–40.5]	[0–92.8]	56	2840	4.8 [4.4–5.2]	95.6 [94.9–96.2]	<0.001
Low risk of bias	8.5 [0.9–21]	[0–65]	5	314	2.7 [1.8–4]	86 [69.5–93.6]	<0.001
Process control	36.8 [18–57.8]	[0–100]	15	1097	6.8 [6–7.7]	97.9 [97.3–98.3]	<0.001
**Treated wastewater**							
Overall	18.0 [9.5–28.3]	[0–83.9]	34	1649	4.7 [4.3–5.2]	95.5 [94.5–96.3]	<0.001
Low risk of bias	16.0 [0–53]	[0–100]	3	92	3.5 [2.2–5.6]	91.8 [79.3–96.8]	<0.001
Process control	18.9 [3.4–41.9]	[0–99.1]	11	826	7 [6.1–8.1]	98 [97.3–98.5]	<0.001
**Surface water**							
Overall	15.0 [10.2–20.6]	[0–68.3]	66	5851	5.2 [4.9–5.6]	96.3 [95.8–96.8]	<0.001
Low risk of bias	28.6 [0–85.7]	[0–100]	3	73	5.1 [3.5–7.5]	96.2 [91.9–98.2]	<0.001
Process control	12.1 [5.9–19.9]	[0–68.4]	33	2620	5.2 [4.7–5.7]	96.3 [95.5–96.9]	<0.001
**Drinking water**							
Overall	0.4 [0–1.8]	[0–6.2]	11	1388	1.7 [1.2–2.3]	64.4 [32–81.3]	0.002
Process control	1.5 [0–17.7]	NA	2	380	2.8 [1.4–5.6]	87.4 [50.7–96.8]	0.005
**Groundwater**							
Overall	2.4 [0.2–6.1]	[0–20.9]	12	2165	3.2 [2.5–3.9]	90 [84.6–93.6]	<0.001
Low risk of bias	0.4 [0–3.3]	NA	2	1158	3 [1.5–5.8]	88.9 [58.1–97.1]	0.003
Process control	1.2 [0.1–3.2]	[0–8.2]	5	618	1.3 [1–2.1]	38.9 [0–77.4]	0.162
**Others**							
Overall	8.5 [3.2–15.7]	[0–50.8]	21	1529	3.7 [3.2–4.3]	92.6 [90–94.5]	<0.001
Process control	0.4 [0–2.7]	[0–7.4]	8	640	1.2 [1–1.9]	34.2 [0–70.9]	0.155

The 95% CI: 95% Confidence Interval. NA: not applicable. H is a measure of the extent of heterogeneity; a value of H > 1 indicates a potential heterogeneity of the prevalence of hepatitis A virus. I^2^ describes the proportion of total variation in prevalence of hepatitis A virus that is due to heterogeneity; values of 25%, 50%, and 75% represent low, moderate, and high heterogeneity, respectively.

## Data Availability

Not applicable.

## Supplementary Materials

The following supporting information can be downloaded at: https://www.mdpi.com/article/10.3390/ijerph20021054/s1.
