# Supplementary material for: Occurrence of Hepatitis A Virus in Water Matrices: A Systematic Review and Meta-Analysis
Source: Int J Environ Res Public Health. 2023 Jan 6;20(2):1054. doi: 10.3390/ijerph20021054 (PMC9859052; doi:10.3390/ijerph20021054)

Study	Positive	Total	Prevalence (%)	95% CI	Weight
**Drinking water**					
Ahmad, 2015_Pakistan_Tap water	0	20	0.00	[0.00; 16.84]	0.5%
Ahmad, 2018_Pakistan_Drinking water	1	44	2.27	[0.06; 12.02]	0.5%
Grabow, 2001_South Africa_Drinking water	12	413	2.91	[1.51; 5.02]	0.5%
Hee, 2006_South Korea_Tap water	0	33	0.00	[0.00; 10.58]	0.5%
Kittigul, 2000_Thailand_Tap water	0	32	0.00	[0.00; 10.89]	0.5%
Kittigul, 2006_Thailand_Tap water	0	100	0.00	[0.00; 3.62]	0.5%
Numanovic, 2004_Bosnia and Herzegovina_Drinking water	0	84	0.00	[0.00; 4.30]	0.5%
Puntaric, 1995_Croatia_Drinking water	7	271	2.58	[1.04; 5.25]	0.5%
Purpari, 2019_Italy_Pipewater	0	11	0.00	[0.00; 28.49]	0.4%
Rigotto, 2010_Brazil_Drinking water	2	24	8.33	[1.03; 27.00]	0.5%
Verma, 2010_India_Drinking water	0	356	0.00	[0.00; 1.03]	0.5%
**Random effect meta-analysis**		**1388**	**0.38**	**[0.00; 1.75]**	**5.6%**
**Prediction interval**	.	.		**[0.00; 6.23]**	---
Heterogeneity: I^2 = 64.4% [32.0%; 81.3%], τ^2 = 0.0041, p = 0.0018					
**Groundwater**					
Abbaszadegan, 1999_United States of America_Groundwater	12	139	8.63	[4.54; 14.59]	0.5%
Bae, 2022_South Korea_Groundwater	0	20	0.00	[0.00; 16.84]	0.5%
Borchardt, 2003_United States of America_Well water	3	194	1.55	[0.32; 4.45]	0.5%
Borchardt, 2004_United States of America_Well water	4	48	8.33	[2.32; 19.98]	0.5%
Carducci, 2003_Italy_Groundwater	0	35	0.00	[0.00; 10.00]	0.5%
De Giglio, 2017_Italy_Well water	0	364	0.00	[0.00; 1.01]	0.5%
De Serres, 1999_United States of America_Well water	6	10	60.00	[26.24; 87.84]	0.4%
Fout, 2003_United States of America_Groundwater	4	321	1.25	[0.34; 3.16]	0.5%
Guerrero−Latorre, 2011_Chad_Wells, Boreholes	0	12	0.00	[0.00; 26.46]	0.4%
Jothikumar, 2000_India_Bore well water samples; well water	6	19	31.58	[12.58; 56.55]	0.4%
Jung, 2011_South Korea_Groundwater	0	39	0.00	[0.00; 9.03]	0.5%
Stokdyk, 2020_United States of America_Well water	0	964	0.00	[0.00; 0.38]	0.5%
**Random effect meta-analysis**		**2165**	**2.36**	**[0.16; 6.09]**	**6.1%**
**Prediction interval**	.	.		**[0.00; 20.90]**	---
Heterogeneity: I^2 = 90% [84.6%; 93.6%], τ^2 = 0.0155, p < 0.0001					
**Others**					
Ahmad, 2016_Pakistan_Drinking water, Sewage sample	12	95	12.63	[6.70; 21.03]	0.5%
Amdiouni, 2017_Morocco_Raw wastewater; Treated effluents	4	50	8.00	[2.22; 19.23]	0.5%
Carducci, 2008_Italy_Sewage samples (entry and exit samples)	2	40	5.00	[0.61; 16.92]	0.5%
Gharbi−Khelifi, 2011_Tunisia_Well−water samples; Drinking water samples	5	10	50.00	[18.71; 81.29]	0.4%
Hassine, 2010_Tunisia_Sewage sample	10	93	10.75	[5.28; 18.89]	0.5%
Hee, 2006_South Korea_Finished water	0	92	0.00	[0.00; 3.93]	0.5%
Janahi, 2020_Bahrain_Influent; Effluent	2	59	3.39	[0.41; 11.71]	0.5%
Jebri, 2012_Tunisia_Raw water; Treated water	0	48	0.00	[0.00; 7.40]	0.5%
Katukiza, 2013_Uganda_Grey water	3	11	27.27	[6.02; 60.97]	0.4%
Nasiri, 2021_Iran_In...uent, before chlorination, e...uent	15	18	83.33	[58.58; 96.42]	0.5%
Peláez, 2016_Colombia_Untreated wastewater; Treated wastewater	77	288	26.74	[21.71; 32.24]	0.5%
Pina, 1998_Spain_Slaughterhouse sewage	0	17	0.00	[0.00; 19.51]	0.5%
Rachida, 2016_South Africa_Irrigation water	8	11	72.73	[39.03; 93.98]	0.4%
Saadoun, 2021_Jordan_Raw sewage; Treated sewage; Surface and pond water	0	33	0.00	[0.00; 10.58]	0.5%
Schvoerer, 2000_France_Treated water, Untreated wastewater	3	13	23.08	[5.04; 53.81]	0.5%
Shin, 2019_South Korea_Irrigation water	0	14	0.00	[0.00; 23.16]	0.5%
Simmons , 2011_United States of America_Wastewater (influent and effluent)	0	30	0.00	[0.00; 11.57]	0.5%
Truchado, 2021_Spain_Sewage effluent; Sewage influent	12	475	2.53	[1.31; 4.37]	0.5%
Tucker, 2000_United States of America_Groundwater, Surface water	1	24	4.17	[0.11; 21.12]	0.5%
Wang, 2018_Sweden_Sewage effluent; Sewage influent	0	12	0.00	[0.00; 26.46]	0.4%
Wang, 2020_South Korea_Irrigation water	0	96	0.00	[0.00; 3.77]	0.5%
**Random effect meta-analysis**		**1529**	**8.53**	**[3.16; 15.69]**	**10.4%**
**Prediction interval**	.	.		**[0.00; 50.79]**	---
Heterogeneity: I^2 = 92.6% [90.0%; 94.5%], τ^2 = 0.0477, p < 0.0001					
**Surface water**					
Ahmad, 2015_Pakistan_Surface water samples	0	30	0.00	[0.00; 11.57]	0.5%
Ahmad, 2018_Pakistan_River water	2	26	7.69	[0.95; 25.13]	0.5%
Aw, 2011_Singapore_Surface water	4	45	8.89	[2.48; 21.22]	0.5%
Bahk, 2020_South Korea_River water samples	4	100	4.00	[1.10; 9.93]	0.5%
Bai, 2019_Philippines_River samples	7	12	58.33	[27.67; 84.83]	0.4%
Borchardt, 2004_United States of America_River water	0	12	0.00	[0.00; 26.46]	0.4%
Carducci, 2006_Italy_River water, Seawater	0	24	0.00	[0.00; 14.25]	0.5%
Chigor, 2012_South Africa_River water	31	72	43.06	[31.43; 55.27]	0.5%
Cioffi, 2021_Italy_Seawater	0	225	0.00	[0.00; 1.63]	0.5%
Corsi, 2014_United States of America_River water	1	63	1.59	[0.04; 8.53]	0.5%
De Paula, 2007_Brazil_River water	48	52	92.31	[81.46; 97.86]	0.5%
de Souza, 2018_Brazil_Streams water	1	84	1.19	[0.03; 6.46]	0.5%
Denis−Mize, 2004_United States of America_Stream water	4	15	26.67	[7.79; 55.10]	0.5%
Dias, 2018_Brazil_Seawater	1	48	2.08	[0.05; 11.07]	0.5%
Divizia, 1993_Italy_River water	9	13	69.23	[38.57; 90.91]	0.5%
Elmahdy, 2016_Brazil_Surface water	14	48	29.17	[16.95; 44.06]	0.5%
Elmahdy, 2016_Brazil_Surface water	13	48	27.08	[15.28; 41.85]	0.5%
Farkas, 2018_United Kingdom_Surface water	0	52	0.00	[0.00; 6.85]	0.5%
Félix, 2010_Mexico_Marine recreational waters	3	32	9.38	[1.98; 25.02]	0.5%
Fernández−Molina, 2004_Spain_Spring water; River water	16	51	31.37	[19.11; 45.89]	0.5%
Fongaro, 2012_Brazil_Surface water	6	48	12.50	[4.73; 25.25]	0.5%
Fongaro, 2019_Brazil_River water	39	45	86.67	[73.21; 94.95]	0.5%
Fumian, 2018_Brazil_River water, Lagoon water	7	32	21.88	[9.28; 39.97]	0.5%
Gersberg, 2006_United States of America_Ocean water samples	11	20	55.00	[31.53; 76.94]	0.5%
Hee, 2006_South Korea_Surface water	0	223	0.00	[0.00; 1.64]	0.5%
Hernandez−Morga, 2009_Mexico_Estuarine water samples	32	40	80.00	[64.35; 90.95]	0.5%
Hot, 2003_France_River water	1	68	1.47	[0.04; 7.92]	0.5%
Iaconelli, 2015_Italy_River water	2	27	7.41	[0.91; 24.29]	0.5%
Iaconelli, 2017_Italy_River water	2	28	7.14	[0.88; 23.50]	0.5%
Jiang, 2004_United States of America_River water	16	21	76.19	[52.83; 91.78]	0.5%
Jones, 2017_Canada_River water	18	1032	1.74	[1.04; 2.74]	0.5%
Katukiza, 2013_Uganda_Surface water	4	26	15.38	[4.36; 34.87]	0.5%
Kittigul, 2000_Thailand_Surface water from canals	3	20	15.00	[3.21; 37.89]	0.5%
Kittigul, 2006_Thailand_Swamps; Canals	9	80	11.25	[5.28; 20.28]	0.5%
Kiulia, 2010_Kenya_River water	8	29	27.59	[12.73; 47.24]	0.5%
Kokkinos, 2018_Greece_Seawater; River water	1	72	1.39	[0.04; 7.50]	0.5%
La Rosa, 2017_Italy_River water	3	32	9.38	[1.98; 25.02]	0.5%
La Rosa, 2021_Italy_Seawater; Discharge water	19	392	4.85	[2.94; 7.47]	0.5%
Lazic, 2015_Serbia_Untreated surface water	0	60	0.00	[0.00; 5.96]	0.5%
Lee, 2008_South Korea_River water	2	58	3.45	[0.42; 11.91]	0.5%
Lee, 2014_South Korea_Surface water	9	265	3.40	[1.56; 6.35]	0.5%
Lenaker, 2017_United States of America_River water	0	290	0.00	[0.00; 1.26]	0.5%
Masachessi, 2018_Argentina_Dam water sample, beaches water sample	11	114	9.65	[4.92; 16.61]	0.5%
Miagostovich, 2014_Brazil_Surface water	0	108	0.00	[0.00; 3.36]	0.5%
Morace, 1993_Italy_River water	8	13	61.54	[31.58; 86.14]	0.5%
Moresco, 2012_Brazil_Seawater	68	132	51.52	[42.66; 60.30]	0.5%
Nasser, 1987_United States of America_Seawater	3	20	15.00	[3.21; 37.89]	0.5%
Ngaosuwankul, 2013_Thailand_Flood water samples	7	100	7.00	[2.86; 13.89]	0.5%
O'Brien, 2017_Uganda_Swamp	8	12	66.67	[34.89; 90.08]	0.4%
Pina, 1998_Spain_River water; Seawater	13	37	35.14	[20.21; 52.54]	0.5%
Pina, 2001_Spain_River water	24	66	36.36	[24.87; 49.13]	0.5%
Prevost, 2015_France_River water, water samples from the tributaries	0	175	0.00	[0.00; 2.09]	0.5%
Purpari, 2019_Italy_Seawater	0	10	0.00	[0.00; 30.85]	0.4%
Pusch, 2005_Germany_Lakeshore	5	38	13.16	[4.41; 28.09]	0.5%
Rachida, 2020_South Africa_Dam water	0	12	0.00	[0.00; 26.46]	0.4%
Rigotto, 2010_Brazil_Lagoon water, Polluted creek, Seawater	5	60	8.33	[2.76; 18.39]	0.5%
Said, 2014_South Africa_Surface water sample	16	18	88.89	[65.29; 98.62]	0.5%
Schvoerer, 2000_France_Bathing water samples	0	26	0.00	[0.00; 13.23]	0.5%
Shaheen, 2019_Egypt_Surface water	11	32	34.38	[18.57; 53.19]	0.5%
Staggemeier, 2017_Brazil_Surface water	0	146	0.00	[0.00; 2.49]	0.5%
Taylor, 2001_South Africa_Surface water	37	102	36.27	[26.98; 46.39]	0.5%
Vantarakis, 1999_Greece_Surface river water; Pond water	0	24	0.00	[0.00; 14.25]	0.5%
Vantarakis, 2005_Greece_Seawater samples	0	234	0.00	[0.00; 1.56]	0.5%
Venter, 2007_South Africa_Surface water	50	308	16.23	[12.30; 20.84]	0.5%
Verma, 2010_India_Dam water, River water	49	73	67.12	[55.13; 77.67]	0.5%
Yanez, 2014_Argentina_River water	5	31	16.13	[5.45; 33.73]	0.5%
**Random effect meta-analysis**		**5851**	**15.04**	**[10.16; 20.59]**	**33.4%**
**Prediction interval**	.	.		**[0.00; 68.31]**	---
Heterogeneity: I^2 = 96.3% [95.8%; 96.8%], τ^2 = 0.0762, p = 0					
**Treated wastewater**					
Adefisoye, 2016_South Africa_Effluent samples	3	48	6.25	[1.31; 17.20]	0.5%
Anastasi, 2008_Italy_Wastewater sample	21	92	22.83	[14.72; 32.75]	0.5%
Aw, 2010_Singapore_Effluent samples	5	18	27.78	[9.69; 53.48]	0.5%
Béji−Hamza, 2014_Tunisia_Treated sewage	46	71	64.79	[52.54; 75.76]	0.5%
Bisseux, 2018_France_Treated sewage	5	26	19.23	[6.55; 39.35]	0.5%
Carducci, 2006_Italy_Sewage effluent	0	12	0.00	[0.00; 26.46]	0.4%
Carducci, 2009_Italy_Effluent sewage	0	30	0.00	[0.00; 11.94]	0.5%
Chacón, 2020_Costa Rica_Effluent wastewater sample	3	58	5.17	[1.08; 14.38]	0.5%
Divizia, 1998_Italy_Treated sewage	3	10	30.00	[6.67; 65.25]	0.4%
Farkas, 2018_United Kingdom_Wastewater effluent	0	39	0.00	[0.00; 9.03]	0.5%
Gcilitshana, 2017_South Africa_Effluent	2	24	8.33	[1.03; 27.00]	0.5%
Iaconelli, 2015_Italy_Treated sewage	3	21	14.29	[3.05; 36.34]	0.5%
Iaconelli, 2017_Italy_Treated effluent, Ultrafiltered effluent	5	33	15.15	[5.11; 31.90]	0.5%
Jehl−Pietri, 1993_France_Sewage effluent	8	13	61.54	[31.58; 86.14]	0.5%
Kamel, 2011_Egypt_Outlet	3	38	7.89	[1.66; 21.38]	0.5%
Kokkinos, 2010_Greece_Outlet	0	26	0.00	[0.00; 13.72]	0.5%
O'Brien, 2017_Uganda_Sewage effluent	9	24	37.50	[18.80; 59.41]	0.5%
Osuolale, 2015_South Africa_Sewage effluent	0	70	0.00	[0.00; 5.13]	0.5%
Ouardani, 2015_Tunisia_Treated sewage influent	92	162	56.79	[48.79; 64.54]	0.5%
Ouardani, 2016_Tunisia_Treated sewage	55	135	40.74	[32.37; 49.53]	0.5%
Petrinca, 2009_Italy_Sewage effluent	16	65	24.62	[14.77; 36.87]	0.5%
Prado, 2011_Brazil_Effluent water	8	22	36.36	[17.20; 59.34]	0.5%
Prado, 2012_Brazil_Treated effluent samples	0	24	0.00	[0.00; 14.25]	0.5%
Prevost, 2015_France_Effluent water	0	100	0.00	[0.00; 3.62]	0.5%
Pusch, 2005_Germany_Sewage plant outflow	11	95	11.58	[5.92; 19.77]	0.5%
Rachida, 2020_South Africa_Treated wastewater discharge	43	52	82.69	[69.67; 91.77]	0.5%
Rodriguez−Manzano, 2012_Spain_Treated sewage sample	3	31	9.68	[2.04; 25.75]	0.5%
Said, 2014_South Africa_Outflow wastewater sample	19	51	37.25	[24.13; 51.92]	0.5%
Schlindwein, 2010_Brazil_Treated wastewater effluent	3	12	25.00	[5.49; 57.19]	0.4%
Steyer, 2015_Slovenia_Sewage effluent	0	12	0.00	[0.00; 26.46]	0.4%
Vaidya, 2002_India_Sewage effluent	2	48	4.17	[0.51; 14.25]	0.5%
Verma, 2010_India_Outlet	0	153	0.00	[0.00; 2.38]	0.5%
Villar, 2007_Brazil_Treated sewage	24	25	96.00	[79.65; 99.90]	0.5%
Wang, 2020_Sweden_Sewage effluent	5	11	45.45	[16.75; 76.62]	0.4%
**Random effect meta-analysis**		**1649**	**18.03**	**[9.53; 28.28]**	**16.9%**
**Prediction interval**	.	.		**[0.00; 83.88]**	---
Heterogeneity: I^2 = 95.5% [94.5%; 96.3%], τ^2 = 0.1110, p < 0.0001					
**Untreated wastewater**					
Ahmad, 2015_Pakistan_Sewage sample	3	20	15.00	[3.21; 37.89]	0.5%
Arankalle, 2006_India_Sewage samples	0	13	0.00	[0.00; 24.71]	0.5%
Aw, 2010_Singapore_Sewage sample	14	18	77.78	[52.36; 93.59]	0.5%
Barrella, 2009_Brazil_Raw sewage sample	24	50	48.00	[33.66; 62.58]	0.5%
Béji−Hamza, 2014_Tunisia_Raw sewage	54	79	68.35	[56.92; 78.37]	0.5%
Bisseux, 2018_France_Raw sewage	16	27	59.26	[38.80; 77.61]	0.5%
Blanco, 2019_Saudi Arabia_Water samples	8	21	38.10	[18.11; 61.56]	0.5%
Carducci, 2006_Italy_Raw sewage	0	12	0.00	[0.00; 26.46]	0.4%
Carducci, 2009_Italy_Influent sewage	0	29	0.00	[0.00; 11.94]	0.5%
Chacón, 2020_Costa Rica_Influent wastewater sample	5	59	8.47	[2.81; 18.68]	0.5%
Chacón, 2021_Costa Rica_Influent (raw, untreated sewage)	5	59	8.47	[2.81; 18.68]	0.5%
Chitambar, 2007_India_Sewage sample	9	10	90.00	[55.50; 99.75]	0.4%
Clemente−Casares, 2003_Spain_Sewage sample	21	27	77.78	[57.74; 91.38]	0.5%
Divizia, 1998_Italy_Raw sewage	8	10	80.00	[44.39; 97.48]	0.4%
Dubrou, 1991_France_Wastewater sample	33	44	75.00	[59.66; 86.81]	0.5%
Farkas, 2018_United Kingdom_Wastewater influent	0	52	0.00	[0.00; 6.85]	0.5%
Formiga−Cruz, 2005_Spain_Raw sewage	2	10	20.00	[2.52; 55.61]	0.4%
Girones, 1995_Spain_Sewage	10	10	100.00	[69.15; 100.00]	0.4%
Gozlan, 2019_Israel_Sewage sample	55	170	32.35	[25.39; 39.94]	0.5%
Gozlan, 2021_Israel_Sewage sample	87	240	36.25	[30.16; 42.68]	0.5%
Iaconelli, 2015_Italy_Raw sewage	7	21	33.33	[14.59; 56.97]	0.5%
Iaconelli, 2017_Italy_Raw sewage	7	21	33.33	[14.59; 56.97]	0.5%
Jehl−Pietri, 1993_France_Sewage affluent	3	13	23.08	[5.04; 53.81]	0.5%
Jothikumar, 1998_United States of America_Raw sewage	2	18	11.11	[1.38; 34.71]	0.5%
Kaas, 2016_New Caledonia (France) _Wastewater	0	12	0.00	[0.00; 26.46]	0.4%
Kamel, 2011_Egypt_Inlet	8	38	21.05	[9.55; 37.32]	0.5%
Kittigul, 2000_Thailand_Sewage	0	48	0.00	[0.00; 7.40]	0.5%
Kiulia, 2010_Kenya_Raw sewage samples	1	13	7.69	[0.19; 36.03]	0.5%
Kokkinos, 2010_Greece_Inlet	1	25	4.00	[0.10; 20.35]	0.5%
Kokkinos, 2011_Greece_Untreated sewage	4	48	8.33	[2.32; 19.98]	0.5%
La Rosa, 2014_Italy_Sewage sample	38	157	24.20	[17.73; 31.67]	0.5%
La Rosa, 2021_Italy_Sewage sample	20	71	28.17	[18.13; 40.10]	0.5%
O'Brien, 2017_Uganda_Sewage influent	11	24	45.83	[25.55; 67.18]	0.5%
Ouardani, 2015_Tunisia_Raw sewage effluent	37	163	22.70	[16.51; 29.90]	0.5%
Ouardani, 2016_Tunisia_Raw sewage	91	136	66.91	[58.33; 74.74]	0.5%
Pellegrinelli, 2019_Italy_Influent	5	33	15.15	[5.11; 31.90]	0.5%
Petrinca, 2009_Italy_Sewage influent	5	27	18.52	[6.30; 38.08]	0.5%
Pietri, 1988_France_Sewage sample	5	15	33.33	[11.82; 61.62]	0.5%
Pina, 1998_Spain_Raw domestic sewage water	4	15	26.67	[7.79; 55.10]	0.5%
Pina, 2001_Spain_Raw sewage sample	31	54	57.41	[43.21; 70.77]	0.5%
Pintó, 2007_Egypt_Raw sewage	25	35	71.43	[53.70; 85.36]	0.5%
Pintó, 2007_Spain_Raw sewage	28	249	11.24	[7.60; 15.84]	0.5%
Prado, 2011_Brazil_Raw sewage	5	12	41.67	[15.17; 72.33]	0.4%
Prado, 2012_Brazil_Raw sewage	14	24	58.33	[36.64; 77.89]	0.5%
Prado, 2021_Brazil_Raw sewage	40	52	76.92	[63.16; 87.47]	0.5%
Rachida, 2020_South Africa_Sewage inflow	43	54	79.63	[66.47; 89.37]	0.5%
Rodriguez−Manzano, 2010_Spain_Sewage influent	7	91	7.69	[3.15; 15.21]	0.5%
Rodriguez−Manzano, 2012_Spain_Raw sewage sample	0	13	0.00	[0.00; 24.71]	0.5%
Thongprachum, 2018_Japan_Raw sewage	0	12	0.00	[0.00; 26.46]	0.4%
Vaidya, 2002_India_Sewage influent	21	86	24.42	[15.80; 34.87]	0.5%
van Zyl, 2019_Kenya_Fecally polluted water samples	59	59	100.00	[93.94; 100.00]	0.5%
Verma, 2010_India_Inlet	0	153	0.00	[0.00; 2.38]	0.5%
Villar, 2007_Brazil_Raw sewage	22	25	88.00	[68.78; 97.45]	0.5%
Wang, 2020_Sweden_Sewage influent	18	26	69.23	[48.21; 85.67]	0.5%
Wong, 2013_United States of America_Untreated sewage sample	0	13	0.00	[0.00; 24.71]	0.5%
Yanez, 2014_Argentina_Raw sewage	5	24	20.83	[7.13; 42.15]	0.5%
**Random effect meta-analysis**		**2840**	**31.47**	**[23.07; 40.48]**	**27.6%**
**Prediction interval**	.	.		**[0.00; 92.83]**	---
Heterogeneity: I^2 = 95.6% [94.9%; 96.2%], τ^2 = 0.1085, p < 0.0001					
**Overall random effect meta-analysis**		**15422**	**16.73**	**[13.40; 20.31]**	**100.0%**
**Prediction interval**				**[0.00; 74.77]**	
Heterogeneity: I^2 = 96.6% [96.3%; 96.8%], τ^2 = 0.0918, p = 0					
Test for subgroup differences: χ^2_5 = 112.24, df = 5 (p < 0.0001)					

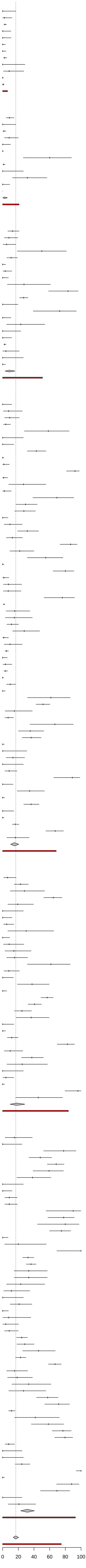

Supplement: Supplementary file 1 [file ijerph-20-01054-s001.zip › Figure S1.pdf]
