# Supplementary material for: Occurrence of Hepatitis A Virus in Water Matrices: A Systematic Review and Meta-Analysis
Source: Int J Environ Res Public Health. 2023 Jan 6;20(2):1054. doi: 10.3390/ijerph20021054 (PMC9859052; doi:10.3390/ijerph20021054)
Supplement: Supplementary file 1 [file ijerph-20-01054-s001.zip › Figure S2.pdf]

Figure S2: Funnel chart for publications of the global hepatitis A virus prevalence in water matrices

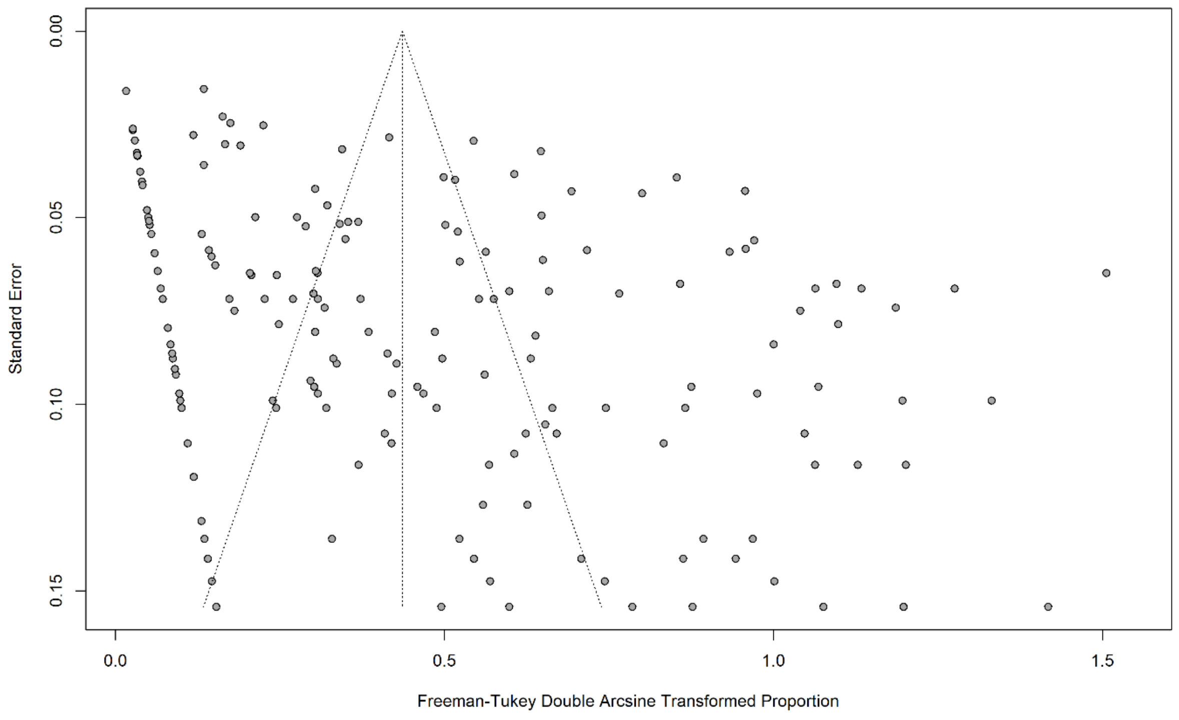

Note: The observation of the asymmetric distribution of the studies on this diagram indicates the existence of publication bias which is confirmed by this P value of the Egger test (P Egger test < 0.001)
