# Supplementary material for: Occurrence of Hepatitis A Virus in Water Matrices: A Systematic Review and Meta-Analysis
Source: Int J Environ Res Public Health. 2023 Jan 6;20(2):1054. doi: 10.3390/ijerph20021054 (PMC9859052; doi:10.3390/ijerph20021054)
Supplement: Supplementary file 1 [file ijerph-20-01054-s001.zip › Supplemental Material - HAV.pdf]

Supplemental Material

Hepatitis A virus in water environments: a systematic review and meta-analysis

Guy Roussel Takuissu [1], Sebastien Kenmoe [2], Jean Thierry Ebogo-Belobo [3], Cyprien Kengne-Ndé [4], Donatien Serge Mbaga [5], Arnol Bowo-Ngandji [5], Juliette Laure Ndzie Ondigui [5], Raoul Kenfack-Momo [6], Serges Tchatchouang [7], Josiane Kenfack-Zanguim [6], Robertine Lontuo Fogang [8], Elisabeth Zeuko'o Menkem [9], Ginette Irma Kame-Ngasse [3], Jeannette Nina Magoudjou-Pekam [6], Carolina Veneri [10], Pamela Mancini [10], Giusy Bonanno Ferraro [10], Marcello Iaconelli [10], Lidia Orlandi [10], Claudia Del Giudice [10], Elisabetta Suffredini [11] and Giuseppina La Rosa [10,*]

[1] Centre for Food, Food Security and Nutrition Research, Institute of Medical Research and Medicinal Plants Studies, Yaounde, Cameroon
[2] Department of Microbiology and Parasitology, University of Buea, Buea, Cameroon
[3] Medical Research Centre, Institute of Medical Research and Medicinal Plants Studies, Yaounde, Cameroon
[4] Epidemiological Surveillance, Evaluation and Research Unit, National AIDS Control Committee, Yaounde, Cameroon
[5] Department of Microbiology, The University of Yaounde I, Yaounde, Cameroon
[6] Department of Biochemistry, The University of Yaounde I, Yaounde, Cameroon
[7] Scientific Direction, Centre Pasteur du Cameroun, Yaounde, Cameroon
[8] Department of Animal Biology, University of Dschang, Dschang, Cameroon
[9] Department of Biomedical Sciences, University of Buea, Buea, Cameroon
[10] Department of Environment and Health, Istituto Superiore di Sanità, 00161 Rome, Italy
[11] Department of Food Safety, Nutrition and Veterinary Public Health, Istituto Superiore di Sanità, 00161 Rome, Italy
* Correspondence: giuseppina.larosa@iss.it

Table of Contents

Table S1: Search strategy

Table S2: Items for risk of bias assessment

Table S3: Main reasons of exclusion of eligible studies

Table S4: Characteristics of included studies

Table S5: Individual characteristics of included studies

Table S6: Risk of bias assessment

Table S7: Detailed results of meta-analysis results for prevalence of hepatitis A virus in various water matrices.

Figure S1: Global prevalence estimate of hepatitis A virus in various water matrices

Figure S2: Funnel chart for publications of the global hepatitis A virus prevalence in water matrices

Table S1: Search strategy

**Search**	**Fields**	
#1	HEV	Hepatitis A virus OR HAV OR Hepatitis A OR Hep A OR Viral hepatitis A OR VHA
#2	Water environments	Water OR "Waste Water" OR Sewage OR Wastewater OR River OR "Surface water" OR Groundwater OR "brackish water" OR Seawater OR "sea water" OR "wastewater treatment plant" OR influent OR effluent OR "drinking water" OR "tap water" OR "potable water" OR lake OR "fresh water" OR freshwater OR "marine water"
#3		#1 AND #2

Table S2: Items for risk of bias assessment

**Modified Hoy et al. tool for cross sectional studies**	**Yes (1)/No (0)**
External validity	

1. Was the study's target population a close representation of the national population in relation to hepatitis A virus prevalence?	**1**
2. Was the sampling frame a true or close representation of the samples?	**1**
3. Was some form of random selection used to select the sample?	**1**
4. Did the author calculate and respect the expected sample size?	**1**
Internal validity	
5. Was an acceptable water matrix definition used in the study?	**1**
6. Was the HAV detection assay shown to have validity and reliability?	**1**
7. Was the same mode of data collection used for all samples?	**1**
8. Was the length of the study period > or = 1 year?	**1**
9. Were the numerator(s) and denominator(s) for the prevalence of hepatitis A virus reported?	**1**
Total score	**9**
**Interpretation of the risk of bias tool**<br>• 7-9: Low risk of bias<br>• 4-6: Moderate risk of bias<br>• 0-3: High risk of bias	

Modified from: Hoy D, Brooks P, Woolf A, Blyth F, March L, Bain C, et al. Assessing risk of bias in prevalence studies: modification of an existing tool and evidence of interrater agreement. J Clin Epidemiol. 2012;65: 934–939. doi:10.1016/j.jclinepi.2011.11.014

Table S3: Main reasons of exclusion of eligible studies

N°	Author, Year	Title	Reason of exclusion
1	Abad, 1994	Disinfection of human enteric viruses in water by copper and silver in combination with low levels of chlorine.	No data on HAV prevalence in water matrices
2	Abbaszadegan, 1993	Evaluation of a microbiological water purifier for inactivation of viruses, bacteria and Giardia cysts.	No data on HAV prevalence in water matrices
3	Abd El Galil, 2004	Combined immunomagnetic separation-molecular beacon-reverse transcription-PCR assay for detection of hepatitis A virus from environmental samples.	Comment on an article
4	Abd el-Galil, 2005	Real-time nucleic acid sequence-based amplification assay for detection of hepatitis A virus.	Comment on an article

5	Abdullah, 1997	Hepatitis A form unsafe water.	Full text or abstract not found
6	Abraira, 2000	A soccer championship and hepatitis A outbreak.	No data on HAV prevalence in water matrices
7	Ahmed, 2020	Prevalence of Waterborne Diseases in Bade, Nguru and Machina Local Government Areas of Yobe State-Nigeria.	No data on HAV prevalence in water matrices
8	Ahmed, 2020	Estimation of hepatitis a virus infection prevalence through drinking water supply of primary schools of Sindh, Pakistan.	No data on HAV prevalence in water matrices
9	Ahmed, 2021	Comparative analysis of rapid concentration methods for the recovery of SARS-CoV-2 and quantification of human enteric viruses and a sewage-associated marker gene in untreated wastewater.	No data on HAV prevalence in water matrices
10	Ali, 2004	Detection of enteric viruses, Giardia and Cryptosporidium in two different types of drinking water treatment facilities.	No data on HAV prevalence in water matrices
11	Ali, 1997	New approaches to virus removal: the way to produce virus-free drinking water.	Full text or abstract not found
12	Ali, 2021	Occurrence of various viruses and recent evidence of SARS-CoV-2 in wastewater systems.	Review
13	Ali., 1997	Ultraviolet, chlorine and ozone disinfection of some enteroviruses in drinking water and treated effluents.	Full text or abstract not found
14	Almeida, 2001	The epidemiology of hepatitis A in Rio de Janeiro: Environmental and domestic risk factors.	No data on HAV prevalence in water matrices
15	Alonso, 2006	Microbiological quality of reclaimed water used for golf courses' irrigation.	No data on HAV prevalence in water matrices
16	Alouini, 1995	Evaluation of an extraction-precipitation method for recovering hepatitis a virus and poliovirus from hardshell clams (Mercenaria mercenaria).	No data on HAV prevalence in water matrices
17	Amado, 2018	A large hepatitis a outbreak in Rio de Janeiro reinforces the need for vaccination of risk groups.	Sample size < or = 10 participants
18	Amado, 2011	Exposure to multiple subgenotypes of hepatitis A virus during an outbreak using matched serum and saliva specimens.	No data on HAV prevalence in water matrices
19	Ambrozic, 2009	Food-borne viruses and food safety considerations.	No data on HAV prevalence in water matrices
20	Anderson-Coughlin, 2021	Enteric Viruses and Pepper Mild Mottle Virus Show Significant Correlation in Select Mid-Atlantic Agricultural Waters.	No data on HAV prevalence in water matrices
21	Anderson-Coughlin, 2019	Recovery and Detection of Enteric Viruses from Non-Traditional Irrigation Water Sources.	No data on HAV prevalence in water matrices
22	Arnal, 1998	Persistence of infectious hepatitis A virus and its genome in artificial seawater.	Full text or abstract not found

23	Arnone, 2007	Waterborne pathogens in urban watersheds.	No data on HAV prevalence in water matrices
24	Arora, 2013	Water borne hepatitis A and hepatitis E in Malwa Region of Punjab, India.	No data on HAV prevalence in water matrices
25	Arraj, 2008	An epidemiological study of enteric viruses in sewage with molecular characterization by RT-PCR and sequence analysis.	No data on HAV prevalence in water matrices
26	Arraj, 2005	Comparison of bacteriophage and enteric virus removal in pilot scale activated sludge plants.	No data on HAV prevalence in water matrices
27	Arvanitidou, 2004	Epidemiological evidence for vaccinating wastewater treatment plant workers against hepatitis A and hepatitis B virus.	No data on HAV prevalence in water matrices
28	Ashbolt, 2004	Microbial contamination of drinking water and disease outcomes in developing regions.	No data on HAV prevalence in water matrices
29	Aziz, 2005	Management of source and drinking-water quality in Pakistan.	Review
30	Báez, 2016	Evidence of the circulation of hepatitis A virus, subgenotype IA, in environmental samples from Antioquia, Colombia.	Article not in English or in French
31	Bagdasarjan, 1979	Human viruses in water, wastewater and soil.	Review
32	Baggi, 2001	Persistence of viral pathogens and bacteriophages during sewage treatment: lack of correlation with indicator bacteria.	No data on HAV prevalence in water matrices
33	Bahcebasi, 1997	Health and the quality of drinking water in Adana City Centre, Turkey.	Full text or abstract not found
34	Balasubramanian, 2016	Enhancing quantitative-PCR-based detection of pathogenic enteric viruses in coastal marine environment using monolithic chromatographic supports.	No data on HAV prevalence in water matrices
35	Barathidasan, 2020	Epidemiological and molecular investigation of a hepatitis A outbreak in Tamil Nadu, Southern India.	No data on HAV prevalence in water matrices
36	Barrett, 2019	Impact of Public Health Interventions on Drinking Water-Associated Outbreaks of Hepatitis A - United States, 1971-2017.	Review
37	Barrimah, 1999	An outbreak of hepatitis A associated with treated waste water used for irrigation.	No data on HAV prevalence in water matrices
38	Batik, 1980	An epidemiologic study of the relationship between hepatitis A and water supply characteristics and treatment.	No data on HAV prevalence in water matrices
39	Bazzardi, 2014	Preliminary Study on Norovirus, Hepatitis A Virus, Escherichia coli and their Potential Seasonality in Shellfish from Different Growing and Harvesting Areas in Sardinia Region.	No data on HAV prevalence in water matrices
40	Belabbes, 1985	Epidemic non-A, non-B viral hepatitis in Algeria: Strong evidence for its spreading by water.	No data on HAV prevalence in water matrices

41	Benabbes, 2013	Norovirus and Other Human Enteric Viruses in Moroccan Shellfish.	No data on HAV prevalence in water matrices
42	Benckert, 2001	[Diagnosis and clinical features of infection with hepatitis A and hepatitis E viruses. Transmission through drinking water and foodstuffs].	Article not in English or in French
43	Berg, 1978	Indicators of viruses in water and food.	Review
44	Bergeisen, 1985	A waterborne outbreak of hepatitis A in Meade County, Kentucky.	No data on HAV prevalence in water matrices
45	Betancourt, 2010	Molecular characterization of sewage-borne pathogens and detection of sewage markers in an urban stream in Caracas, Venezuela.	No data on HAV prevalence in water matrices
46	Bigoraj, 2014	Occurrence of Norovirus and Hepatitis A Virus in Wild Mussels Collected from the Baltic Sea.	No data on HAV prevalence in water matrices
47	Biziagos, 1988	Long-term survival of hepatitis A virus and poliovirus type 1 in mineral water.	No data on HAV prevalence in water matrices
48	Biziagos, 1989	Hepatitis A virus concentration from experimentally contaminated distilled, tap, waste and seawater.	No data on HAV prevalence in water matrices
49	Blaise-Boisseau, 2010	Duplex real-time qRT-PCR for the detection of hepatitis A virus in water and raspberries using the MS2 bacteriophage as a process control.	No data on HAV prevalence in water matrices
50	Bloch, 1990	Recovery of hepatitis A virus from a water supply responsible for a common source outbreak of hepatitis A.	No data on HAV prevalence in water matrices
51	Blokhin, 1992	Improvement of the method of gathering of hepatitis A viruses and its evaluation by water analysis.	Article not in English or in French
52	Borgmästars, 2017	Improved Detection of Norovirus and Hepatitis A Virus in Surface Water by Applying Pre-PCR Processing.	Sample size < or = 10 participants
53	Borgmästars, 2021	Comparison of Skimmed Milk and Lanthanum Flocculation for Concentration of Pathogenic Viruses in Water.	Sample size < or = 10 participants
54	Bosch, 1993	Disinfection of human enteric viruses in water by copper: Silver and reduced levels of chlorine.	No data on HAV prevalence in water matrices
55	Bosch, 1991	Concentration of hepatitis A virus in environmental samples.	Not possible to extract data on HAV prevalence
56	Bosch, 2008	New tools for the study and direct surveillance of viral pathogens in water.	Review
57	Bosch, 2001	Human enteric viruses in Coquina clams associated with a large hepatitis A outbreak.	No data on HAV prevalence in water matrices
58	Bowen, 1983	Hepatitis A associated with a hardware store water fountain and a contaminated well in Lancaster County, Pennsylvania, 1980.	No data on HAV prevalence in water matrices
59	Boxman, 2016	International linkage of two food-borne hepatitis a clusters through traceback of mussels, The Netherlands, 2012.	No data on HAV prevalence in water matrices

60	Bozaykut, 2012	Environmental risk factors for hepatitis a.	No data on HAV prevalence in water matrices
61	Braga, 2008	[Estimation of risk areas for hepatitis A].	Article not in English or in French
62	Brake, 2018	Spatial and Temporal Distribution of Norovirus and E. coli in Sydney Rock Oysters Following a Sewage Overflow into an Estuary.	No data on HAV prevalence in water matrices
63	Brassard, 2011	Simultaneous recovery of bacteria and viruses from contaminated water and spinach by a filtration method.	No data on HAV prevalence in water matrices
64	Brassard, 2005	Concentration and detection of hepatitis A virus and rotavirus in spring water samples by reverse transcription-PCR.	No data on HAV prevalence in water matrices
65	Brisebois, 2018	Human viral pathogens are pervasive in wastewater treatment center aerosols.	No data on HAV prevalence in water matrices
66	Brooks, 2005	Detection and quantification of hepatitis A virus in seawater via real-time RT-PCR.	No data on HAV prevalence in water matrices
67	Brown, 2000	Investigations of the epidemiology of infections with hepatitis A virus in Jamaica.	No data on HAV prevalence in water matrices
68	Brunkard, 2011	Surveillance for waterborne disease outbreaks associated with drinking water - united states, 2007-2008.	No data on HAV prevalence in water matrices
69	Bryan, 1974	An outbreak of hepatitis A associated with recreational lake water.	No data on HAV prevalence in water matrices
70	Buehler, 1984	Hepatitis A, a day care home and a contaminated well.	Full text or abstract not found
71	Butot, 2004	Attachment of enteric viruses to bottles.	No data on HAV prevalence in water matrices
72	Cabezas Sánchez, 2018	Infectious diseases related to water in Peru.	Article not in English or in French
73	Cadamuro, 2021	Enteric viruses in lentic and lotic freshwater habitats from Brazil's Midwest and South regions in the Guarani Aquifer area.	Not possible to extract data on HAV prevalence
74	Callahan, 1995	Comparative survival of hepatitis a virus, poliovirus and indicator viruses in geographically diverse seawaters.	No data on HAV prevalence in water matrices
75	Cannella, 2010	Detection of human enteric viruses in shellfish, vegetables, waters and environmental samples: A preliminary study.	Not possible to extract data on HAV prevalence
76	Casas, 2002	Detection of enteroviruses, hepatitis a virus and rotaviruses in sewage by means of an immunomagnetic capture reverse transcription-PCR assay.	Sample size < or = 10 participants
77	Cecuk, 1993	Human viruses in the costal environment of a Croatian harbour.	Full text or abstract not found

78	Célia da Silva Lanna, 2019	Household-based biodigesters promote reduction of enteric virus and bacteria in vulnerable and poverty rural area.	No data on HAV prevalence in water matrices
79	Centers for Disease Control and Prevention, 1997	Hepatitis A associated with consumption of frozen strawberries--Michigan, March 1997.	No data on HAV prevalence in water matrices
80	Centers for Disease Control and Prevention, 2011	Establishment of a viral hepatitis surveillance system--Pakistan, 2009-2011.	No data on HAV prevalence in water matrices
81	Chadha, 2009	Outbreaks of hepatitis A among children in western India.	Sample size < or = 10 participants
82	Chakraborty, 1982	Non-A non-B viral hepatitis: A common-source outbreak traced to sewage contamination of drinking water.	Full text or abstract not found
83	Chang, 1997	Detection of hepatitis A virus in environmental samples by coupled reverse transcription and polymerase chain reaction.	No data on HAV prevalence in water matrices
84	Chen, 2011	[A hepatitis A outbreak caused by contaminated well water in a primary school of Jiangxi province, China, 2009].	Article not in English or in French
85	Chesnot, 2012	Investigating emerging microbiological risks in local river water used by water treatment plants in the Paris metropolitan area.	No data on HAV prevalence in water matrices
86	Chigor, 2014	Assessment of the Risks for Human Health of Adenoviruses, Hepatitis A Virus, Rotaviruses and Enteroviruses in the Buffalo River and Three Source Water Dams in the Eastern Cape.	No data on HAV prevalence in water matrices
87	Chobe, 2009	Investigation of a hepatitis A outbreak from Shimla Himachal Pradesh.	Sample size < or = 10 participants
88	Christiansen, 1957	A water-borne hepatitis epidemic.	Full text or abstract not found
89	Chung, 1993	Comparative survival of indicator viruses and enteric viruses in seawater and sediment.	No data on HAV prevalence in water matrices
90	Clark, 1982	An environmental health survey of drinking water contamination by leachate from a pesticide waste dump in Hardeman County, Tennessee.	No data on HAV prevalence in water matrices
91	Clarke, 1970	Viruses occurring in water.	Full text or abstract not found
92	Clarke, 1964	HUMAN ENTERIC VIRUSES IN SEWAGE.	Full text or abstract not found
93	Cliver, 1985	Vehicular transmission of hepatitis A.	Full text or abstract not found
94	Cliver, 2010	Early days of food and environmental virology.	Review
95	Coelho, 2003	Hepatitis A virus detection in oysters (Crassostrea gigas) in Santa Catarina State, Brazil, by reverse transcription-polymerase chain reaction.	No data on HAV prevalence in water matrices
96	Cormier, 2016	Concentration and detection of hepatitis A virus and its indicator from artificial seawater using zeolite.	No data on HAV prevalence in water matrices
97	Coudray-Meunier, 2015	A comparative study of digital RT-PCR and RT-qPCR for quantification of Hepatitis A virus and Norovirus in lettuce and water samples.	No data on HAV prevalence in water matrices

98	Coudray-Meunier, 2013	Discrimination of infectious hepatitis A virus and rotavirus by combining dyes and surfactants with RT-qPCR.	No data on HAV prevalence in water matrices
99	Courault, 2017	Assessment and risk modeling of airborne enteric viruses emitted from wastewater reused for irrigation.	No data on HAV prevalence in water matrices
100	Crance, 1998	Effect of temperature on the survival of hepatitis A virus and its capsidal antigen in synthetic seawater.	No data on HAV prevalence in water matrices
101	Craun, 1985	A summary of waterborne illness transmitted through contaminated groundwater.	No data on HAV prevalence in water matrices
102	Crilly, 2019	Spatial and temporal analysis of Hepatitis A cases in fier district, Albania, 1986–2016.	No data on HAV prevalence in water matrices
103	Croci, 2000	Determination of enteroviruses, hepatitis A virus, bacteriophages and Escherichia coli in Adriatic Sea mussels.	No data on HAV prevalence in water matrices
104	Croci, 2007	Assessment of human enteric viruses in shellfish from the northern Adriatic sea.	No data on HAV prevalence in water matrices
105	Cruvinel, 2019	Waterborne diseases in waste pickers of Estrutural, Brazil, the second largest open-air dumpsite in world.	No data on HAV prevalence in water matrices
106	Cuevas-Ferrando, 2020	Assessment of ISO Method 15216 to Quantify Hepatitis E Virus in Bottled Water.	No data on HAV prevalence in water matrices
107	Cuevas-Ferrando, 2020	HEV Occurrence in Waste and Drinking Water Treatment Plants.	No data on HAV prevalence in water matrices
108	D'Ugo, 2016	Detection of Human Enteric Viruses in Freshwater from European Countries.	No data on HAV prevalence in water matrices
109	D'Amico, 1978	Epidemiological investigations on hepatitis A and B diffusion during an outbreak in a Sicilian town in conditions of high incidence of infections.	Article not in English or in French
110	Davidkin, 2007	Molecular epidemiology of hepatitis A in St. Petersburg, Russia, 1997-2003.	No data on HAV prevalence in water matrices
111	De Abreu Corrêa, 2012	Stability of human enteric viruses in seawater samples from mollusc depuration tanks coupled with ultraviolet irradiation.	No data on HAV prevalence in water matrices
112	De Leon, 1991	Detection of rotaviruses in water by gene probes.	No data on HAV prevalence in water matrices
113	Deng, 1974	Detection of hepatitis A virus in environmental samples by antigen-capture PCR.	Sample size < or = 10 participants
114	Denis, 1980	Control of virus contamination of water.	Full text or abstract not found

115	Di Maria, 1981	Epidemic outbreak of type A viral hepatitis in a town in the province of Florence (Rufina).	No data on HAV prevalence in water matrices
116	Di Pasquale, 2010	Comparison of different concentration methods for the detection of hepatitis A virus and calicivirus from bottled natural mineral waters.	No data on HAV prevalence in water matrices
117	Di Pinto, 2004	A comparison of RT-PCR-based assays for the detection of HAV from shellfish.	No data on HAV prevalence in water matrices
118	Dilawari, 1994	Hepatitis E virus: epidemiological, clinical and serological studies of north Indian epidemic.	No data on HAV prevalence in water matrices
119	Ding, 1982	An outbreak of water-borne infection by viral hepatitis A.	Article not in English or in French
120	Divizia, 2008	Sewage workers: Risk of acquiring enteric virus infections including Hepatitis A.	No data on HAV prevalence in water matrices
121	Divizia, 1989	HAV recovery from tap water: evaluation of different types of membranes.	No data on HAV prevalence in water matrices
122	Divizia, 1989	Isolation of wild-type hepatitis A virus from the environment.	Sample size < or = 10 participants
123	Divizia, 2005	Nucleotide correlation between HAV isolates from human patients and environmental samples.	Sample size < or = 10 participants
124	Divizia, 1993	Hepatitis A virus identification in an outbreak by enzymatic amplification.	Sample size < or = 10 participants
125	Dubois, 2007	Detection and quantification by real-time RT-PCR of hepatitis A virus from inoculated tap waters, salad vegetables, and soft fruits: Characterization of the method performances.	No data on HAV prevalence in water matrices
126	Dudarev, 2013	Food and water security issues in Russia III: food- and waterborne diseases in the Russian Arctic, Siberia and the Far East, 2000-2011.	Review
127	Durmisević, 1998	Status of the quality of drinking water provided to schools in the area of the Zenoj-Doboj Canton.	Article not in English or in French
128	Eifan, 2013	Enteric viruses and aquatic environment.	Review
129	El Esnawy, 1998	Detection of hepatitis E virus in greater Cairo two wastewater treatment plants and its prevalence among workers of these plants.	Full text or abstract not found
130	El-Esnawy, 1998	Detection of hepatitis E virus in greater Cairo. Two wastewater treatment plants and its prevalence among workers of these plants.	No data on HAV prevalence in water matrices
131	Elkana, 1996	Study of the association between waterborne diseases and microbial water quality in Israel.	Review
132	Elkana, 1983	Detection of hepatitis A virus in sewage.	Not possible to extract data on HAV prevalence
133	Elmahdy, 2022	Detection of Norovirus and Hepatitis A Virus in Strawberry and Green Leafy Vegetables by Using RT-qPCR in Egypt.	No data on HAV prevalence in water matrices
134	El-Senousy, 2017	Assessment and Evaluation of an Integrated Hybrid Anaerobic–Aerobic Sewage Treatment System for the Removal of Enteric Viruses.	No data on HAV prevalence in water matrices

135	Enriquez, 1995	Survival of the enteric adenoviruses 40 and 41 in tap, sea, and waste water.	No data on HAV prevalence in water matrices
136	Enriquez, 1992	Accumulation and persistence of hepatitis A virus in mussels.	No data on HAV prevalence in water matrices
137	Errezola Saizar, 1983	Epidemiologic study of a water-borne outbreak of hepatitis A.	Full text or abstract not found
138	Espigares, 1999	Detection of hepatitis A virus in wastewater.	Sample size < or = 10 participants
139	Fang, 2017	[Studies and application method and quality control system for detecting hepatitis A virus in water for fruits and vegetables production].	Article not in English or in French
140	Farkas, 2017	Evaluation of Two Triplex One-Step qRT-PCR Assays for the Quantification of Human Enteric Viruses in Environmental Samples.	No data on HAV prevalence in water matrices
141	Farquhar, 1952	Epidemic of viral hepatitis apparently spread by drinking water and by contact.	No data on HAV prevalence in water matrices
142	Fattal, 1983	The prevalence of viral hepatitis and other enteric disease in communities utilizing wastewater in agriculture.	No data on HAV prevalence in water matrices
143	Fernandez-Cassi, 2018	Metagenomics for the study of viruses in urban sewage as a tool for public health surveillance.	No data on HAV prevalence in water matrices
144	Flehmig, 1988	[Isolation of infectious hepatitis A viruses from clearing sludge].	Article not in English or in French
145	Fongaro, 2015	Human and animal enteric virus in groundwater from deep wells, and recreational and network water.	Sample size < or = 10 participants
146	Fusco, 2019	Detection of Hepatitis A Virus and Other Enteric Viruses in Shellfish Collected in the Gulf of Naples, Italy.	No data on HAV prevalence in water matrices
147	Gajardo, 1991	Adsorption-elution with negatively and positively-charged glass powder for the concentration of hepatitis A virus from water.	No data on HAV prevalence in water matrices
148	Gammie, 1997	Does hepatitis A pose a significant health risk to recreational water users?	No data on HAV prevalence in water matrices
149	Gaon, 1975	Evaluation of the secondary attack rate in families with viral hepatitis: a three year study in former year district of Sarajevo, Yugoslavia.	No data on HAV prevalence in water matrices
150	Gaon, 1982	Characteristics of water-borne epidemics of viral hepatitis in Yugoslavia.	No data on HAV prevalence in water matrices
151	Gargano, 2017	Mortality from selected diseases that can be transmitted by water - United States, 2003-2009.	No data on HAV prevalence in water matrices
152	Garin, 1994	Exposure to enteroviruses and hepatitis A virus among divers in environmental waters in France, first biological and serological survey of a controlled cohort.	No data on HAV prevalence in water matrices

153	Gavan, 1970	An epidemic of waterborne infectious hepatitis in France.	No data on HAV prevalence in water matrices
154	Geetharani, 2016	Hepatitis A outbreak due to contaminated public water in Tiruchirappalli Corporation, Tamil Nadu, India, 2015.	No data on HAV prevalence in water matrices
155	Gerba, 1988	Enteric virus: Risk assessment of ocean disposal of sewage sludge.	No data on HAV prevalence in water matrices
156	Gerba, 1989	Application of gene probes to virus detection in water.	No data on HAV prevalence in water matrices
157	Gerba, 1988	Enterovirus detection in water with gene probes.	No data on HAV prevalence in water matrices
158	Getahun, 2015	Hepatitis A outbreak in Ba subdivision, Fiji, October–December 2013.	No data on HAV prevalence in water matrices
159	Gharbi-khelifi, 2007	A 1-year study of the epidemiology of hepatitis A virus in Tunisia.	Not possible to extract data on HAV prevalence
160	Gilgen, 1997	Three-step isolation method for sensitive detection of enterovirus, rotavirus, hepatitis A virus, and small round structured viruses in water samples.	Sample size < or = 10 participants
161	Girones, 1989	Natural inactivation of enteric viruses in seawater.	No data on HAV prevalence in water matrices
162	Giwa, 2020	Dissecting microbial community structure in sewage treatment plant for pathogens' detection using metagenomic sequencing technology.	No data on HAV prevalence in water matrices
163	González-Saldía, 2019	Fecal pollution source tracking and thalassogenic diseases: The temporal-spatial concordance between maximum concentrations of human mitochondrial DNA in seawater and Hepatitis A outbreaks among a coastal population.	No data on HAV prevalence in water matrices
164	Goswami, 1993	Detection of hepatitis A virus in Mercenaria mercenaria by coupled reverse transcription and polymerase chain reaction.	No data on HAV prevalence in water matrices
165	Gozlan, 2018	Hepatitis A outbreak in men having sex with men in Israel 2017: Environmental and clinical surveillance.	No data on HAV prevalence in water matrices
166	Grabow, 1983	Inactivation of hepatitis A virus and indicator organisms in water by free chlorine residuals.	No data on HAV prevalence in water matrices
167	Grabow, 1985	Inactivation of hepatitis A virus, other enteric viruses and indicator organisms in water by chlorination.	No data on HAV prevalence in water matrices
168	Grabow, 1989	Selection of indicator systems for human viruses in polluted seawater and shellfish.	No data on HAV prevalence in water matrices
169	Grabow, 1978	Studies on the isolation and identification of hepatitis viruses in water.	Not possible to extract data on HAV prevalence

170	Graff, 1993	Detection of hepatitis A virus in sewage sludge by antigen capture polymerase chain reaction.	Sample size < or = 10 participants
171	Green, 1995	Enzymatic amplification of enteric viruses from wastewaters.	No data on HAV prevalence in water matrices
172	Green, 1999	Comparative detection of enteric viruses in wastewaters, sediments and oysters by reverse transcription-PCR and cell culture.	Not possible to extract data on HAV prevalence
173	Griffin, 2003	Pathogenic human viruses in coastal waters.	Review
174	Griffin, 1999	Detection of viral pathogens by reverse transcriptase PCR and of microbial indicators by standard methods in the canals of the Florida Keys.	Not possible to extract data on HAV prevalence
175	Guajardo-Leiva, 2020	Metagenomic insights into the sewage RNA virosphere of a large city.	No data on HAV prevalence in water matrices
176	Gupta, 2019	Epidemiological investigation of an acute viral hepatitis outbreak in an urbanized rural area in a North Indian Union Territory.	No data on HAV prevalence in water matrices
177	Guzmán, 2015	[Quality of water for human consumption and its association with morbimortality in Colombia, 2008-2012].	No data on HAV prevalence in water matrices
178	Hamner, 2006	The role of water use patterns and sewage pollution in incidence of water-borne/enteric diseases along the Ganges River in Varanasi, India.	No data on HAV prevalence in water matrices
179	Hau, 1999	Prevalence of enteric hepatitis A and E viruses in the Mekong River delta region of Vietnam.	No data on HAV prevalence in water matrices
180	Helcl, 1990	Epidemics of water-borne viral hepatitis type A in the CR.	Full text or abstract not found
181	Hellmér, 2014	Detection of pathogenic viruses in sewage provided early warnings of hepatitis A virus and norovirus outbreaks.	No data on HAV prevalence in water matrices
182	Hennechart-Collette, 2020	Evaluation of three different filters and two methods for recovering viruses from drinking water.	No data on HAV prevalence in water matrices
183	Hennechart-Collette, 2021	Detection of norovirus, hepatitis A and hepatitis E viruses in multicomponent foodstuffs.	No data on HAV prevalence in water matrices
184	Hennechart-Collette, 2015	Determination of which virus to use as a process control when testing for the presence of hepatitis A virus and norovirus in food and water.	No data on HAV prevalence in water matrices
185	Herbold, 1989	Comparison of ozone inactivation, in flowing water, of hepatitis A virus, poliovirus 1, and indicator organisms.	No data on HAV prevalence in water matrices
186	Hernroth, 2002	Environmental factors influencing human viral pathogens and their potential indicator organisms in the blue mussel, Mytilus edulis: The first Scandinavian report.	No data on HAV prevalence in water matrices
187	Herwaldt, 1991	Waterborne-disease outbreaks, 1989-1990.	No data on HAV prevalence in water matrices

188	Hewitt, 2006	Effect of heat treatment on hepatitis A virus and norovirus in New Zealand Greenshell mussels (Perna canaliculus) by quantitative real-time reverse transcription PCR and cell culture.	No data on HAV prevalence in water matrices
189	Hirani, 2013	Characterization of effluent water qualities from satellite membrane bioreactor facilities.	No data on HAV prevalence in water matrices
190	Hossain, 2009	Hepatitis E outbreak in a low income urban community in Bangladesh.	No data on HAV prevalence in water matrices
191	Hrdy, 2021	MOL-PCR and xMAP Technology: A Multiplex System for Fast Detection of Food- and Waterborne Viruses.	No data on HAV prevalence in water matrices
192	Hughes, 1975	Outbreaks of waterborne disease in the United States, 1973.	No data on HAV prevalence in water matrices
193	Huguet, 2012	A comparison of different concentration methods for the detection of viruses present in bottled waters and those adsorbed to water bottle surfaces.	No data on HAV prevalence in water matrices
194	Imagawa, 2010	Detection waterborne diseases associated viruses in the river water Metro Manila and Bulacan, the Philippines.	Not possible to extract data on HAV prevalence
195	Ishii, 2011	Epidemiological and genetic analysis of a diffuse outbreak of hepatitis A in Japan, 2010.	No data on HAV prevalence in water matrices
196	Ishii, 2014	Microfluidic quantitative PCR for simultaneous quantification of multiple viruses in environmental water samples.	No data on HAV prevalence in water matrices
197	Jaykus, 1993	Application of RT-PCR for the detection of enteric viruses in oysters.	No data on HAV prevalence in water matrices
198	Jean, 2001	Detection of hepatitis A virus by the nucleic acid sequence-based amplification technique and comparison with reverse transcription-PCR.	No data on HAV prevalence in water matrices
199	Jiang, 1986	Detection of hepatitis A virus in seeded estuarine samples by hybridization with cDNA probes.	No data on HAV prevalence in water matrices
200	Jiang, 1987	Detection of hepatitis A virus by hybridization with single-stranded RNA probes.	No data on HAV prevalence in water matrices
201	Jiménez-Moleón, 2011	Waterborne diseases in the state of Mexico, Mexico (2000-2005).	No data on HAV prevalence in water matrices
202	John, 2006	Large outbreak of hepatitis A in Kerala [1].	No data on HAV prevalence in water matrices
203	Jothikumar, 2010	Development of an RNA extraction protocol for detection of waterborne viruses by reverse transcriptase quantitative PCR (RT-qPCR).	No data on HAV prevalence in water matrices
204	Junaid, 2014	Sanitary Survey of Drinking Water Quality in Plateau State, Nigeria.	No data on HAV prevalence in water matrices

205	Kaas, 2019	Detection of Human Enteric Viruses in French Polynesian Wastewaters, Environmental Waters and Giant Clams.	Sample size < or = 10 participants
206	Kadri, 2018	Hepatitis A and E outbreak surveillance during 2015–2017 in Kashmir, India: Is the water to blame?	No data on HAV prevalence in water matrices
207	Kaevska, 2016	Changes in Microbial Composition of Wastewater During Treatment in a Full-Scale Plant.	No data on HAV prevalence in water matrices
208	Kanaaneh, 1995	A Human Rights Approach for Access to Clean Drinking Water: A Case Study.	Case report
209	Kankaria, 2020	Epidemiological investigation of an outbreak of Acute Viral Hepatitis A and E in a semi-urban locality in Chandigarh, North Indian Union Territory, 2016-17.	No data on HAV prevalence in water matrices
210	Karasaki, 2012	Hepatitis A virus contamination in the river and soil of Kitakyushu area in Japan.	No data on HAV prevalence in water matrices
211	Katoch, 2016	Outbreak investigation of acute viral hepatitis in Kangra valley, Himachal Pradesh, India, 2014-2015.	No data on HAV prevalence in water matrices
212	Katzenelson, 1976	Risk of communicable disease infection associated with wastewater irrigation in agricultural settlements.	No data on HAV prevalence in water matrices
213	Kaur, 2017	Hepatitis E virus: A leading cause of waterborne viral hepatitis in Northwest Districts of Punjab, India.	No data on HAV prevalence in water matrices
214	Khan, 2018	Prevalent fecal contamination in drinking water resources and potential health risks in Swat, Pakistan.	No data on HAV prevalence in water matrices
215	Khan, 2007	Environmental health indicators in New Zealand: Drinking water - A case study.	No data on HAV prevalence in water matrices
216	Khan, 2014	HAV in fresh vegetables: a hidden health risk in district Mardan, Pakistan.	No data on HAV prevalence in water matrices
217	Kim, 2006	Detection of enteroviruses and mammalian reoviruses in Korean environmental waters.	No data on HAV prevalence in water matrices
218	Kingsley, 2001	Rapid and efficient extraction method for reverse transcription-PCR detection of hepatitis A and Norwalk-like viruses in shellfish.	No data on HAV prevalence in water matrices
219	Kingsley, 2003	Persistence of hepatitis A virus in oysters.	No data on HAV prevalence in water matrices
220	Kojouharova, 2006	Current outbreak of hepatitis A in Bulgaria, 2006.	Review
221	Kokkinos, 2017	Virological Quality of Irrigation Water in Leafy Green Vegetables and Berry Fruits Production Chains.	No data on HAV prevalence in water matrices
222	Kokkinos, 2010	Molecular characterization of hepatitis a virus isolates from environmental and clinical samples in Greece.	No data on HAV prevalence in water matrices

223	Kokkinos, 2011	Molecular detection of multiple viral targets in untreated urban sewage from Greece.	Duplicates
224	Kollins, 1966	The presence of human enteric viruses in sewage and their removal by conventional sewage treatment methods.	Review
225	Komar, 1992	Administration of water-soluble vitamins in viral hepatitis A.	No data on HAV prevalence in water matrices
226	Kotwal, 2014	Environmental persistence and transfer of enteric viruses.	Review
227	Kovac, 2009	A novel method for concentrating hepatitis A virus and caliciviruses from bottled water.	No data on HAV prevalence in water matrices
228	Kozísek, 2009	[Waterborne diseases outbreaks in the Czech Republic, 1995-2005].	No data on HAV prevalence in water matrices
229	Krasil'nikov, 1985	[Isolation of viruses from water using porous silica].	No data on HAV prevalence in water matrices
230	Kratochvil, 1998	Quality assessment of drinking water in Prague in relation to the incidence of diarrhoeal diseases.	No data on HAV prevalence in water matrices
231	Kretschmer, 1972	Water and sewage as transmission factors in viral hepatitis, demonstrated by practical examples.	Article not in English or in French
232	Krikelis, 1985	Detection of indigenous enteric viruses in raw sewage effluents of the city of Athens, Greece, during a two year survey.	No data on HAV prevalence in water matrices
233	Kueh, 1989	Recovery of viruses and bacteria in waters off Bondi beach: a pilot study.	No data on HAV prevalence in water matrices
234	Kukavica-Ibrulj, 2003	Immunofluorescent detection and quantitation of hepatitis A virus in sewage treatment effluent and on agri-food surfaces using scanning confocal microscopy.	No data on HAV prevalence in water matrices
235	Kumar, 2016	Hepatitis A outbreak associated with unsafe drinking water in a medical college student's hostel, New Delhi, India, 2014.	No data on HAV prevalence in water matrices
236	Kumar, 2015	Viral Hepatitis Surveillance--India, 2011-2013.	Review
237	La Bella, 2017	Food-Borne Viruses in Shellfish: Investigation on Norovirus and HAV Presence in Apulia (SE Italy).	No data on HAV prevalence in water matrices
238	La Rosa, 2012	Emerging and potentially emerging viruses in water environments.	Review
239	Le Guyader, 2000	Three-year study to assess human enteric viruses in shellfish.	No data on HAV prevalence in water matrices
240	Le Guyader, 1995	Use of RT seminested PCR to assess viral contamination in caribbean rivers (martinique).	No data on HAV prevalence in water matrices
241	Leblanc, 2019	Persistence of murine norovirus, bovine rotavirus, and hepatitis A virus on stainless steel surfaces, in spring water, and on blueberries.	No data on HAV prevalence in water matrices

242	Lee, 2013	Development of a new cell culture-based method and optimized protocol for the detection of enteric viruses.	No data on HAV prevalence in water matrices
243	Leisinger, 2013	Use of silica as a carrier to recover and prepare waterborne enteric viruses for detection by RT-PCR.	No data on HAV prevalence in water matrices
244	Leshchuk, 1996	Concentration of hepatitis A and B virus antigens using water-soluble polymers.	No data on HAV prevalence in water matrices
245	Lewis, 1988	Polyethylene glycol precipitation for recovery of pathogenic viruses, including hepatitis A virus and human rotavirus, from oyster, water, and sediment samples.	No data on HAV prevalence in water matrices
246	Li, 1998	A new and simple method for concentration of enteric viruses from water.	No data on HAV prevalence in water matrices
247	Li, 2009	Detection of enteroviruses and hepatitis a virus in water by consensus primer multiplex RT-PCR.	No data on HAV prevalence in water matrices
248	Lin, 2019	Concentration of hepatitis A virus from mimicked water samples by membrane filtration method.	Article not in English or in French
249	Linglof, 191	An epidemic of water-borne hepatitis A.	Article not in English or in French
250	Lipp, 2007	Analysis of multiple enteric viral targets as sewage markers in coral reefs.	Not possible to extract data on HAV prevalence
251	López-Gálvez, 2016	Occurrence of enteric viruses in reclaimed and surface irrigation water: relationship with microbiological and physicochemical indicators.	Not possible to extract data on HAV prevalence
252	Luksamijarulkul, 1994	Microbiological quality of drinking water and using water of a Chao Phya River community, Bangkok.	No data on HAV prevalence in water matrices
253	Lund, 1982	Waterborne virus diseases.	No data on HAV prevalence in water matrices
254	Macaluso, 2021	Occurrence of Human Enteric Viruses in Shellfish along the Production and Distribution Chain in Sicily, Italy.	No data on HAV prevalence in water matrices
255	Maksimov, 2003	[Indications of pathogenic viruses in water of Lake Baikal and its tributaries].	Article not in English or in French
256	Mamontova, 2000	[Viral pollution of drinking water in industrial towns of Eastern Siberia].	No data on HAV prevalence in water matrices
257	Manja, 1982	A simple field test for the detection of faecal pollution in drinking water.	No data on HAV prevalence in water matrices
258	Manor, 2017	Evidence for Hepatitis A virus endemic circulation in Israel despite universal toddlers' vaccination since 1999 and low clinical incidence in all age groups.	No data on HAV prevalence in water matrices
259	Manso, 2010	Genotyping of hepatitis A virus detected in bivalve shellfish in Galicia (NW Spain).	No data on HAV prevalence in water matrices

260	Marcos , 2014	Detección del virus de la Hepatitis A en dos muestras de agua del Río Sechura, usando transcriptasa-reversa PCR en tiempo real.	Article not in English or in French
261	Marie, 2017	Viruses in the environment - presence and diversity of bacteriophage and enteric virus populations in the Umhlangane River, Durban, South Africa.	No data on HAV prevalence in water matrices
262	Martínez Amate, 2010	Acute toxic hepatitis due to drinking water.	No data on HAV prevalence in water matrices
263	Masciopinto, 2019	Human health risk assessment for the occurrence of enteric viruses in drinking water from wells: Role of flood runoff injections.	No data on HAV prevalence in water matrices
264	Masciopinto, 2011	Analytical solution for the modeling of the natural time-dependent reduction of waterborne viruses injected into fractured aquifers.	No data on HAV prevalence in water matrices
265	Masclaux, 2014	Assessment of airborne virus contamination in wastewater treatment plants.	No data on HAV prevalence in water matrices
266	Mayr, 1980	Viruses in drinking water - an underestimated danger?	Article not in English or in French
267	McCall, 2020	Identification of multiple potential viral diseases in a large urban center using wastewater surveillance.	Not possible to extract data on HAV prevalence
268	McCall, 2021	Assessment of enteric viruses during a hepatitis outbreak in Detroit MI using wastewater surveillance and metagenomic analysis.	No data on HAV prevalence in water matrices
269	Meinick, 1982	Viruses in surface and drinking waters.	Review
270	Melnick, 1978	Viruses in water.	No data on HAV prevalence in water matrices
271	Mendelson, 2015	Endemic circulation of hepatitis a virus in israel in spite of universal vaccination program evidenced by clinical and environmental surveillance.	No data on HAV prevalence in water matrices
272	Meng, 2008	Development of an extraction and concentration method for the detection of hepatitis A virus in different samples.	No data on HAV prevalence in water matrices
273	Metcalf, 1988	Detection of hepatitis A virus in estuarine samples by gene probe assay.	Review
274	Metcalf, 1995	Environmental virology: From detection of virus in sewage and water by isolation to identification by molecular biology - A trip of over 50 years.	Review
275	Metcalf, 1972	The occurrence of human viruses and coliphage in marine waters and shellfish.	No data on HAV prevalence in water matrices
276	Ming Yi, 1995	Persistence of inoculated hepatitis A virus in mixed human and animal wastes.	No data on HAV prevalence in water matrices
277	Mirzaei, 2016	Health-related Microbial Quality of Drinking Water in Kangavar, Western Iran.	No data on HAV prevalence in water matrices

278	Momba, 2009	Survival of somatic and F-RNA coliphages in treated wastewater effluents and their impact on viral quality of the receiving water bodies in the Eastern Cape Province-South Africa.	No data on HAV prevalence in water matrices
279	Monceyron, 1994	Detection of hepatitis A virus in clinical and environmental samples by immunomagnetic separation and PCR.	No data on HAV prevalence in water matrices
280	Montuori, 2009	Wastewater workers and hepatitis A virus infection.	No data on HAV prevalence in water matrices
281	Moore, 1993	Surveillance for waterborne disease outbreaks--United States, 1991-1992.	No data on HAV prevalence in water matrices
282	Morace, 2002	Microbial quality of wastewater: Detection of hepatitis A virus by reverse transcriptase-polymerase chain reaction.	No data on HAV prevalence in water matrices
283	Moreno, 2009	Phylogenetic analysis indicates human origin of rotavirus and hepatitis A virus strains found in the drinking water of western Colombia.	Article not in English or in French
284	Morgado, 1982	An outbreAk of hepatitis a apparently associated with use of contaminated water.	Full text or abstract not found
285	Mosley, 1937	Infectious hepatitis; report of an outbreak probably caused by drinking water.	No data on HAV prevalence in water matrices
286	Mphaka, 2016	Hepatitis A virus outbreak in a compound in Tshwane district, Gauteng, South Africa: October 2014-March 2015.	No data on HAV prevalence in water matrices
287	Murhekar, 2018	Epidemiology of hepatitis a and Hepatitis E based on laboratory surveillance data-India, 2014-2017.	No data on HAV prevalence in water matrices
288	Muscillo, 1997	Enteric virus detection in Adriatic seawater by cell culture, polymerase chain reaction and polyacrylamide gel electrophoresis.	No data on HAV prevalence in water matrices
289	Myrmel, 2006	Enteric viruses in inlet and outlet samples from sewage treatment plants.	No data on HAV prevalence in water matrices
290	Nabil-Ben-Salem, 2012	Evolutionary pattern of 5'-UTR of enteroviruses and primer update for the detection of enteroviral RNA in environmental samples.	No data on HAV prevalence in water matrices
291	Nasheri, 2021	Survival and Inactivation by Advanced Oxidative Process of Foodborne Viruses in Model Low-Moisture Foods.	No data on HAV prevalence in water matrices
292	Nasser, 1994	Prevalence and fate of hepatitis A virus in water.	Review
293	Nasser, 1991	Detection of human rotaviruses in fresh and estuarine waters by dot-blot hybridization.	No data on HAV prevalence in water matrices
294	Nasser, 1999	Quantitative assessment of the inactivation of pathogenic and indicator viruses in natural water sources.	No data on HAV prevalence in water matrices

295	Nasser, 1993	Comparative survival of E. coli, F+bacteriophages, HAV and poliovirus 1 in wastewater and groundwater.	No data on HAV prevalence in water matrices
296	Nechiporenko, 1986	Use of natural sorbents for isolation of enteroviruses and hepatitis A virus from water bodies.	Article not in English or in French
297	Nedachin, 2015	WASTE WATERS AS THE RESERVOIR OF INTESTINAL ENTERIC VIRAL INFECTIONS.	Article not in English or in French
298	Nedachin, 1993	Effect of various chlorine doses on inactivation of hepatitis A virus in water.	Article not in English or in French
299	Nedachin, 1993	[Rapid method for detecting hepatitis A virus antigen in the water].	Article not in English or in French
300	Nieuwenhuijse, 2017	Metagenomic sequencing for surveillance of food- and waterborne viral diseases.	Review
301	Nikiforov, 1974	A water-borne outbreak of infectious hepatitis.	Article not in English or in French
302	Normann, 1995	Molecular epidemiology of an outbreak of hepatitis A in Italy.	No data on HAV prevalence in water matrices
303	Novaković, 1983	An epidemic of viral hepatitis A after consumption of mussels from polluted seawater.	Article not in English or in French
304	Oh, 2009	Development of RT-PCR method to detect various human enteric viruses.	Article not in English or in French
305	Okoh, 2010	Inadequately treated wastewater as a source of human enteric viruses in the environment.	Review
306	Orive, 2020	Early SARS-CoV-2 outbreak detection by sewage-based epidemiology.	Review
307	Orlova, 2005	Dynamics of viral hepatitis A morbidity in Minsk.	Article not in English or in French
308	Oshiki, 2018	Microfluidic PCR amplification and MiSeq amplicon sequencing techniques for high-throughput detection and genotyping of human pathogenic RNA viruses in human feces, sewage, and oysters.	Sample size < or = 10 participants
309	Palombi, 2001	Tirane, Albania: Survey on drinking water quality and facilities.	No data on HAV prevalence in water matrices
310	Pan, 2015	High-throughput screening assay for the environmental water samples using cellular response profiles.	No data on HAV prevalence in water matrices
311	Pana, 1987	Isolation of hepatitis A virus from polluted river water on FRP/3 cells.	Sample size < or = 10 participants
312	Park, 2010	Human enteric viruses in groundwater.	No data on HAV prevalence in water matrices
313	Parshionikar, 2004	Development of homologous viral internal controls for use in RT-PCR assays of waterborne enteric viruses.	No data on HAV prevalence in water matrices
314	Parshionikar, 2004	Development of homologous viral internal controls for use in RT-PCR assays of waterborne enteric viruses.	No data on HAV prevalence in water matrices

315	Patti, 1987	Hepatitis A virus and poliovirus 1 inactivation in estuarine water.	No data on HAV prevalence in water matrices
316	Pavoni, 2022	Detection of Hepatitis A Virus and Norovirus in Different Food Categories: A 6-Year Survey in Italy.	No data on HAV prevalence in water matrices
317	Peczenik, 1956	An apparently water-borne outbreak of infectious hepatitis.	No data on HAV prevalence in water matrices
318	Perelle, 2009	Use of a robotic RNA purification protocol based on the NucliSens® easyMAG™ for real-time RT-PCR detection of hepatitis A virus in bottled water.	No data on HAV prevalence in water matrices
319	Pérez-Méndez, 2014	Concentration of enteric viruses from tap water using an anion exchange resin-based method.	No data on HAV prevalence in water matrices
320	Phanuwan, 2006	Monitoring of human enteric viruses and coliform bacteria in waters after urban flood in Jakarta, Indonesia.	Not possible to extract data on HAV prevalence
321	Pietri, 1988	Demonstration of hepatitis A virus (HAV) in sewage by immune electron microscopy.	Full text or abstract not found
322	Pietri, 1988	Hepatitis A virus levels in shellfish exposed in a natural marine environment to the effluent from a treated sewage outfall.	Not possible to extract data on HAV prevalence
323	Pietri, 1987	Detection of hepatitis A virus in experimentally contaminated seawater using a radio-immunological test: Sensitivity of the method.	No data on HAV prevalence in water matrices
324	Pinheiro, 2015	INTERMEDIATE ENDEMICITY OF HEPATITIS A VIRUS INFECTION IN RURAL SETTLEMENT PROJECTS OF SOUTHWEST GOIÁS, BRAZIL.	No data on HAV prevalence in water matrices
325	Polo, 2018	Hepatitis A Virus Disinfection in Water by Solar Photo–Fenton Systems.	No data on HAV prevalence in water matrices
326	Pommepuy, 2011	Monitoring and modeling the microbiological quality of water and shellfish.	Review
327	Pontrelli, 2008	Epidemiological and virological characterization of a large community-wide outbreak of hepatitis A in southern Italy.	No data on HAV prevalence in water matrices
328	Poonawagul, 1995	Outbreak of hepatitis A in a college traced to contaminated water reservoir in cafeteria.	No data on HAV prevalence in water matrices
329	Prado, 2013	The efficiency of concentration methods used to detect enteric viruses in anaerobically digested sludge.	No data on HAV prevalence in water matrices
330	Prado, 2014	Detection of enteric viruses in activated sludge by feasible concentration methods.	No data on HAV prevalence in water matrices
331	Prado, 2013	The efficiency of concentration methods used to detect enteric viruses in anaerobically digested sludge.	No data on HAV prevalence in water matrices
332	Prevot, 1993	Detection of human hepatitis A virus in environmental water by an antigen-capture polymerase chain reaction method.	No data on HAV prevalence in water matrices

333	Prez, 2018	Tracking enteric viruses in green vegetables from central Argentina: potential association with viral contamination of irrigation waters.	No data on HAV prevalence in water matrices
334	Primavesi, 1963	Virus infections and their significance in water hygiene.	Article not in English or in French
335	Purnell, 2016	Removal of phages and viral pathogens in a full-scale MBR: Implications for wastewater reuse and potable water.	No data on HAV prevalence in water matrices
336	Purpari, 2018	Detection of human enteric viruses from shellfish, vegetable and water samples collected in Sicily.	Duplicates
337	Pusch, 2005	Detection of enteric viruses and bacterial indicators in German environmental waters.	Not possible to extract data on HAV prevalence
338	Puvacić, 1977	Epidemiological and clinical circumstances of contracting viral hepatitis through drinking water.	Article not in English or in French
339	Qiao, 2016	Comparison of concentration methods for detection of hepatitis A virus in water samples.	No data on HAV prevalence in water matrices
340	Qing-Ling, 2008	Development of an extraction and concentration method for the detection of hepatitis A virus in different samples.	No data on HAV prevalence in water matrices
341	Rakesh, 2014	Investigating a community-wide outbreak of hepatitis a in India.	No data on HAV prevalence in water matrices
342	Rakesh, 2018	Investigating a community wide outbreak of hepatitis A in Kerala, India.	No data on HAV prevalence in water matrices
343	Ramia, 1985	Transmission of viral infections by the water route: implications for developing countries.	No data on HAV prevalence in water matrices
344	Randazzo, 2019	Interlaboratory Comparative Study to Detect Potentially Infectious Human Enteric Viruses in Influent and Effluent Waters.	No data on HAV prevalence in water matrices
345	Randazzo, 2017	Improving efficiency of viability-qPCR for selective detection of infectious HAV in food and water samples.	No data on HAV prevalence in water matrices
346	Randel, 1962	Infectious hepatitis: a waterborne outbreak at an air base in France.	No data on HAV prevalence in water matrices
347	Rao, 1981	Detection of viruses in drinking water by concentration on magnetic iron oxide.	No data on HAV prevalence in water matrices
348	Rao , 1986	Human viruses in sediments, sludges, and soils.	No data on HAV prevalence in water matrices
349	Raveendran, 2016	Investigation of an Outbreak of Hepatitis A in a Coastal Area, Kerala, Southern India.	No data on HAV prevalence in water matrices
350	Rawal, 1964	EPIDEMIOLOGY OF WATER BORNE INFECTIOUS HEPATITIS IN A LOCALITY IN POONA.	Full text or abstract not found

351	Reynolds, 2001	ICC/PCR detection of enteroviruses and hepatitis A virus in environmental samples.	No data on HAV prevalence in water matrices
352	Rigotto, 2009	Evaluation of HA negatively charged membranes in the recovery of human adenoviruses and hepatitis A virus in different water matrices.	No data on HAV prevalence in water matrices
353	Rohayem, 2006	Assessing the risk of transmission of viral diseases in flooded areas: Viral load of the river Elbe in Dresden during the flood of August 2002.	No data on HAV prevalence in water matrices
354	Romalde, 1996	New molecular methods for the detection of hepatitis A and Norwalk viruses in shellfish.	No data on HAV prevalence in water matrices
355	Rose, 2006	Quantitation of hepatitis A virus and enterovirus levels in the lagoon canals and Lido beach of Venice, Italy, using real-time RT-PCR.	No data on HAV prevalence in water matrices
356	Rosenberg, 1980	The risk of acquiring hepatitis from sewage-contaminated water.	No data on HAV prevalence in water matrices
357	Rowe, 2009	Hepatitis a outbreak epidemiologically linked to a food handler in Melbourne, Victoria.	No data on HAV prevalence in water matrices
358	Ruchusatsawat , 2016	An Outbreak of Acute Hepatitis Caused by Genotype IB Hepatitis A Viruses Contaminating the Water Supply in Thailand.	No data on HAV prevalence in water matrices
359	Rumler, 2000	Prevalence of hepatitis A in sewage and drain pipe constructors.	No data on HAV prevalence in water matrices
360	Rusinol, 2020	Microbiological contamination of conventional and reclaimed irrigation water: Evaluation and management measures.	No data on HAV prevalence in water matrices
361	Rusinol, 2020	Metagenomic analysis of viruses, bacteria and protozoa in irrigation water.	No data on HAV prevalence in water matrices
362	Ryu, 2019	Hepatitis a virus infection from a contaminated tap of ground water facility in a Neighborhood Park, Republic of Korea.	No data on HAV prevalence in water matrices
363	Rzezutka, 2004	Survival of human enteric viruses in the environment and food.	Review
364	Salcerini-Pierangeli, 1966	Epidemiological aspects of viral hepatitis in the Upper Valley of the Tiber River.	No data on HAV prevalence in water matrices
365	Schastnyi, 1988	Multiyear and seasonal hepatitis A morbidity in different population age groups in certain areas.	No data on HAV prevalence in water matrices
366	Schlenker, 1999	Incidence rates of hepatitis A by ZIP code area, Salt Lake County, Utah, 1992-1996.	No data on HAV prevalence in water matrices
367	Schlindwein, 2009	Comparative study of two extraction methods for enteric virus recovery from sewage sludge by molecular methods.	No data on HAV prevalence in water matrices

368	Schlosser, 1995	Viral hepatitis A and exposure to sewage: a confirmed occupational risk.	Full text or abstract not found
369	Schnattinger, 1985	Detection of hepatitis A virus in drinking water by enzyme-immunoassay using ultracentrifugation for virus concentration.	Selection of samples with already HAV result known
370	Schultz, 2011	Collaborative validation of a rapid method for efficient virus concentration in bottled water.	No data on HAV prevalence in water matrices
371	Schuster, 2005	Infectious disease outbreaks related to drinking water in Canada, 1974-2001.	No data on HAV prevalence in water matrices
372	Schwab, 1993	Development of PCR methods for enteric virus detection in water.	No data on HAV prevalence in water matrices
373	Schwab, 1995	Concentration and purification of beef extract mock eluates from water samples for the detection of enteroviruses, hepatitis A virus, and Norwalk virus by reverse transcription-PCR.	No data on HAV prevalence in water matrices
374	Scoglio, 1989	Water quality and microbiological status of the distribution system: traditional parameters and emerging parameters.	No data on HAV prevalence in water matrices
375	Sedmak, 2005	Nine-year study of the occurrence of culturable viruses in source water for two drinking water treatment plants and the influent and effluent of a wastewater treatment plant in Milwaukee, Wisconsin (August 1994 through July 2003).	No data on HAV prevalence in water matrices
376	Sedyaningsih-Mamahit, 2002	First documented outbreak of hepatitis E virus transmission in Java, Indonesia.	No data on HAV prevalence in water matrices
377	Shahin, 2018	Hepatitis A outbreak traced back to ice used in fruit juice in Nellikuzhi panchayat, Ernakulam district, Kerala state.	No data on HAV prevalence in water matrices
378	Shasheen, 2022	Quantitative RT-PCR detection of human noroviruses and hepatitis A virus in fresh produce and surface water used for irrigation in the Mansoura and Giza regions, Egypt.	No data on HAV prevalence in water matrices
379	Shieh, 1991	Detection of hepatitis A virus and other enteroviruses in water by ssRNA probes.	No data on HAV prevalence in water matrices
380	Shin, 2017	A waterborne outbreak involving hepatitis A virus genotype IA at a residential facility in the Republic of Korea in 2015.	Not possible to extract data on HAV prevalence
381	Shuval, 2003	Estimating the global burden of thalassogenic diseases: human infectious diseases caused by wastewater pollution of the marine environment.	Review
382	Sidenko, 1962	Data on detecting antigens (viruses) in water after washing the hands.	Article not in English or in French
383	Sidhu, 2013	Sewage pollution in urban stormwater runoff as evident from the widespread presence of multiple microbial and chemical source tracking markers.	No data on HAV prevalence in water matrices
384	Sincero, 2006	Detection of hepatitis A virus (HAV) in oysters (Crassostrea gigas).	No data on HAV prevalence in water matrices

385	Sinclair, 2009	Viruses in recreational water-borne disease outbreaks: A review.	Review
386	Singh, 2021	Microbiological status of drinking water sources and its relationship with human health in Solan, India.	No data on HAV prevalence in water matrices
387	Singh, 2006	Epidemiological investigation of an outbreak of viral hepatitis.	No data on HAV prevalence in water matrices
388	Smith, 1979	Viruses in water.	Editorial
389	Sobsey, 1985	Evaluation of methods for concentrating hepatitis A virus from drinking water.	No data on HAV prevalence in water matrices
390	Sobsey, 1985	Detection of hepatitis A virus (HAV) in drinking water.	No data on HAV prevalence in water matrices
391	Sobsey, 1991	Comparative inactivation of hepatitis A virus and other enteroviruses in water by iodine.	No data on HAV prevalence in water matrices
392	Sorensen, 2021	Seasonality of enteric viruses in groundwater-derived public water sources.	Not possible to extract data on HAV prevalence
393	Soule, 2000	Ultrafiltration and reverse transcription-polymerase chain reaction: An efficient process for poliovirus, rotavirus and hepatitis A virus detection in water.	No data on HAV prevalence in water matrices
394	Souza, 2012	Evaluation of tropical water sources and mollusks in southern Brazil using microbiological, biochemical, and chemical parameters.	Sample size < or = 10 participants
395	Souza, 2018	Low occurrence of Hepatitis A virus in water samples from an urban area of Southern Brazil.	No data on HAV prevalence in water matrices
396	Sowmyanarayanan, 2008	Investigation of a hepatitis A outbreak in children in an urban slum in Vellore, Tamil Nadu, using geographic information systems.	No data on HAV prevalence in water matrices
397	Springthorpe, 2001	Comparison of static and dynamic disinfection models for bacteria and viruses in water of varying quality.	No data on HAV prevalence in water matrices
398	Springthorpe, 1993	In situ survival of indicator bacteria, MS-2 phage and human pathogenic viruses in river water.	No data on HAV prevalence in water matrices
399	Sree Kalpana, 2016	Hepatitis E outbreak among factory workers due to contaminated factory water, Mandya District, Karnataka, India, 2015.	No data on HAV prevalence in water matrices
400	Straub, 1994	Detection of naturally occurring enteroviruses and hepatitis A virus in undigested and anaerobically digested sludge using the polymerase chain reaction.	Sample size < or = 10 participants
401	Subrahmanyan, 1977	Persistence of enteroviruses in sewage sludge.	No data on HAV prevalence in water matrices
402	Suffredini, 2008	Occurrence of enteric viruses in shellfish and relation to climatic-environmental factors.	No data on HAV prevalence in water matrices

403	Sundkvist, 2000	Outbreak of hepatitis A spread by contaminated drinking glasses in a public house.	No data on HAV prevalence in water matrices
404	Sutmoller, 1982	A water-borne hepatitis A outbreak in Rio de Janeiro.	No data on HAV prevalence in water matrices
405	Symonds, 2009	Eukaryotic viruses in wastewater samples from the United States.	No data on HAV prevalence in water matrices
406	Tani, 1992	Enteric virus levels in river water.	No data on HAV prevalence in water matrices
407	Tauil, 2010	Hepatite a outbreak in an urban area of Luziânia, state of Goiás, Brazil, 2009.	Article not in English or in French
408	Taylor, 1995	A serosurvey of water-borne pathogens amongst canoeists in South Africa.	No data on HAV prevalence in water matrices
409	Telniceanu, 1971	Epidemiological aspects of viral hepatitis. Living conditions and water transmission.	Full text or abstract not found
410	Thornton, 1995	The risk of hepatitis A from sewage contamination of a water supply.	No data on HAV prevalence in water matrices
411	Torok, 2021	Investigation of F-RNA Bacteriophage as a Tool in Re-Opening Australian Oyster Growing Areas Following Sewage Spills.	No data on HAV prevalence in water matrices
412	Tripathy, 2019	Study of a hepatitis e virus outbreak involving drinking water and sewage contamination in Shimla, India, 2015-2016.	Sample size < or = 10 participants
413	Tripathy, 2021	An outbreak of hepatitis E in Yavatmal, India, 2019.	Sample size < or = 10 participants
414	Trudel-Ferland, 2021	Persistence of Hepatitis A Virus RNA in Water, on Non-porous Surfaces, and on Blueberries.	No data on HAV prevalence in water matrices
415	Tsai, 1993	Simple method of concentrating enteroviruses and hepatitis a virus from sewage and ocean water for rapid detection by reverse transcriptase- polymerase chain reaction.	Sample size < or = 10 participants
416	Tsai, 1994	Detection of poliovirus, hepatitis A virus, and rotavirus from sewage and ocean water by triplex reverse transcriptase PCR.	Sample size < or = 10 participants
417	Tulchinsky, 2000	Safety of community drinking-water and outreaks of waterborne enteric disease: Israel, 1976-97.	No data on HAV prevalence in water matrices
418	Umesha, 2008	Prevalence of human pathogenic enteric viruses in bivalve molluscan shellfish and cultured shrimp in south west coast of India.	No data on HAV prevalence in water matrices
419	Upfold, 2021	Occurrence of Human Enteric Viruses in Water Sources and Shellfish: A Focus on Africa.	Review
420	Vasil'ev, 2006	The evaluation of contamination of water objects with hepatitis A virus (HAV) and the impact of HAV on morbidity trends in large seaports of the Ukraine.	Article not in English or in French
421	Venugopalan, 2004	Hepatitis A outbreak in Hulu Langat District, Selangor State, Malaysia during April--October 2002.	No data on HAV prevalence in water matrices

422	Vilim, 1977	Viral hepatitis A: water epidemic in a bungalow community (Czech).	Article not in English or in French
423	Villamizar, 2021	Identification of enteric viruses from raw water using fluoro-immuno-magnetic separation coupled to RT-PCR.	No data on HAV prevalence in water matrices
424	Villar, 2002	Hepatitis A Outbreak in a Public School in Rio de Janeiro, Brazil.	No data on HAV prevalence in water matrices
425	Villar, 2006	Evaluation of methods used to concentrate and detect hepatitis A virus in water samples.	Sample size < or = 10 participants
426	Vishwakarma, 2021	Aptamer-based approaches for the detection of waterborne pathogens.	Review
427	Vonstille, 1993	Hepatitis A epidemics from utility sewage in Ocoee, Florida.	No data on HAV prevalence in water matrices
428	Wallender, 2014	Contributing factors to disease outbreaks associated with untreated groundwater.	No data on HAV prevalence in water matrices
429	Walter, 1985	Interactions between biotic and abiotic factors and viruses in a water system.	No data on HAV prevalence in water matrices
430	Wang, 2018	Differential removal of human pathogenic viruses from sewage by conventional and ozone treatments.	No data on HAV prevalence in water matrices
431	Wanke, 1987	Viral hepatitis and gastroenteritis transmitted by shellfish and water.	Review
432	Warlen, 1998	Hepatitis A in waste water treatment plant workers: Is vaccination necessary? [2].	Full text or abstract not found
433	Weldon, 2000	Prevalence of antibody to hepatitis a virus in drinking water workers and wastewater workers in Texas from 1996 to 1997.	No data on HAV prevalence in water matrices
434	Whatley, 1968	A waterborne outbreak of infectious hepatitis in a small Maryland town.	No data on HAV prevalence in water matrices
435	Williamson, 2011	Enteric viruses in New Zealand drinking-water sources.	No data on HAV prevalence in water matrices
436	Yang, 1993	A new method of RNA preparation for detection of hepatitis A virus in environmental samples by the polymerase chain reaction.	No data on HAV prevalence in water matrices
437	Yang, 2011	Major human Hepatitis A virus genotype in Hong Kong marine waters and detection by real-time PCR.	Sample size < or = 10 participants
438	Yassin, 2006	Assessment of microbiological water quality and its relation to human health in Gaza Governorate, Gaza Strip.	No data on HAV prevalence in water matrices
439	Ye-Qing, 2012	An outbreak of hepatitis A associated with a contaminated well in a middle school, Guangxi, China.	No data on HAV prevalence in water matrices
440	Yoder, 2008	Surveillance for waterborne disease and outbreaks associated with drinking water and water not intended for drinking--United States, 2005-2006.	No data on HAV prevalence in water matrices

441	Yoon, 2009	Epidemiological and virological characterization of a communityoutbreak of hepatitis A: Hospital-based case control study.	No data on HAV prevalence in water matrices
442	Yousuf, 2014	Survey of Gram Negative and Gram Positive Bacteria in Drinking Water Supplies in Karachi, Pakistan.	No data on HAV prevalence in water matrices
443	Yue, 1993	Pollution of Inshore Seawater and Shellfish Due to Hepatitis A Virus in Liaoning Province.	Full text or abstract not found
444	Zak, 1988	Evaluation of the role of a water factor in the seasonal incidence of hepatitis A.	Article not in English or in French
445	Zhang, 2009	An outbreak of hepatitis A in recently vaccinated students from ice snacks made from contaminated well water.	No data on HAV prevalence in water matrices
446	Zhang, 2009	Detection and quantification of hepatitis a virus in Tianjin Coastal Seawater of Bohai Bay.	Article not in English or in French
447	Zhenzhu, 1993	Drinking Water Quality and Intestinal Infectious Diseases in Urban and Rural Boarding Schools in Guangxi.	Full text or abstract not found
448	Zhou, 1991	Concentration and detection of hepatitis A virus and rotavirus from shellfish by hybridization tests.	No data on HAV prevalence in water matrices
449	Zlobina, 1993	The role of the water factor of transmission in the spread of viral hepatitis A and dysentery under the conditions in an industrial city.	Article not in English or in French
450	Zoni, 2006	[Integrate cell culture--PCR (ICC/PCR) in viruses researches in environmental and food samples. Note I].	Article not in English or in French

Table S4: Characteristics of included studies

**Characteristics**	**Overall n=200** (%)	**Untreated wastewater n=56** (%)	**Treated wastewater n=34** (%)	**Surface water n=66** (%)	**Drinking water n=11** (%)	**Groundwate r n=12** (%)	**Others n=21** (%)
**Year of publication; range**	1987-2022	1991-2021	1993-2021	1987-2021	1995-2019	1999-2022	2000-2021
**Period of sampling; range**	1986-2020	1996-2018	2004-2018	1986-2020	1991-2017	1995-2014	1998-2019
**Starting volume for concentration (mL); range**	16.5-1000000	20-20000	16.5-20000	100-20000	1000-1000000	1500-100000	100-10000
**Concentration data (genome copies /L); range**	<1 to 3.7×10^{10}	<1 to 3.7×10^{10}	2.3×10^{1} to 3.3×10^{7}	1.5×10^{1} to 10^{7}			6.87×10^{-1} to 7.4×10^{-1}
**Sampling**							
Non probabilistic	195 (97.5)	56 (100)	34 (100)	64 (97.0)	11 (100)	10 (83.3)	20 (95.2)
Probabilistic	5 (2.5)			2 (3.0)		2 (16.7)	1 (4.8)
**Number of sites**							
Multicenter	130 (65.0)	31 (55.4)	22 (64.7)	53 (80.3)	6 (54.6)	8 (66.7)	10 (47.6)
Monocenter	69 (34.5)	24 (42.9)	12 (35.3)	13 (19.7)	5 (45.5)	4 (33.3)	11 (52.4)
Nationally representative	1 (0.5)	1 (1.8)					
**Timing of samples collection**							
Prospectively	197 (98.5)	55 (98.2)	33 (97.1)	66 (100)	11 (100)	12 (100)	20 (95.2)
Retrospectively	2 (1.0)		1 (2.9)				1 (4.8)
Retroprospectively	1 (0.5)	1 (1.8)					
**Countries**							
Italy	29 (14.5)	9 (16.1)	7 (20.6)	9 (13.6)	1 (9.1)	2 (16.7)	1 (4.8)
Brazil	22 (11.0)	5 (8.9)	4 (11.8)	12 (18.2)	1 (9.1)		

**Characteristics**	**Overall n=200** (%)	**Untreated wastewater n=56** (%)	**Treated wastewater n=34** (%)	**Surface water n=66** (%)	**Drinking water n=11** (%)	**Groundwater n=12** (%)	**Others n=21** (%)
United States of America	17 (8.5)	2 (3.6)		7 (10.6)		6 (50.0)	2 (9.5)
Spain	14 (7.0)	8 (14.3)	1 (2.9)	3 (4.6)			2 (9.5)
South Africa	13 (6.5)	1 (1.8)	5 (14.7)	5 (7.6)	1 (9.1)		1 (4.8)
France	11 (5.5)	4 (7.1)	3 (8.8)	3 (4.6)			1 (4.8)
South Korea	10 (5.0)			4 (6.1)	1 (9.1)	2 (16.7)	3 (14.3)
India	9 (4.5)	4 (7.1)	2 (5.9)	1 (1.5)	1 (9.1)	1 (8.3)	
Tunisia	9 (4.5)	3 (5.4)	3 (8.8)				3 (14.3)
Greece	6 (3.0)	2 (3.6)	1 (2.9)	3 (4.6)			
Pakistan	6 (3.0)	1 (1.8)		2 (3.0)	2 (18.2)		1 (4.8)
Thailand	6 (3.0)	1 (1.8)		3 (4.6)	2 (18.2)		
Uganda	5 (2.5)	1 (1.8)	1 (2.9)	2 (3.0)			1 (4.8)
Egypt	4 (2.0)	2 (3.6)	1 (2.9)	1 (1.5)			
Argentina	3 (1.5)	1 (1.8)		2 (3.0)			
Costa Rica	3 (1.5)	2 (3.6)	1 (2.9)				
Kenya	3 (1.5)	2 (3.6)		1 (1.5)			
Singapore	3 (1.5)	1 (1.8)	1 (2.9)	1 (1.5)			
Sweden	3 (1.5)	1 (1.8)	1 (2.9)				1 (4.8)
United Kingdom	3 (1.5)	1 (1.8)	1 (2.9)	1 (1.5)			
Germany	2 (1.0)		1 (2.9)	1 (1.5)			
Israel	2 (1.0)	2 (3.6)					
Mexico	2 (1.0)			2 (3.0)			
Bahrain	1 (0.5)						1 (4.8)
Bosnia and Herzegovina	1 (0.5)				1 (9.1)		
Canada	1 (0.5)			1 (1.5)			
Chad	1 (0.5)					1 (8.3)	
Colombia	1 (0.5)						1 (4.8)
Croatia	1 (0.5)				1 (9.1)		
Iran	1 (0.5)						1 (4.8)
Japan	1 (0.5)	1 (1.8)					

Characteristics	Overall n=200 (%)	Untreated wastewater n=56 (%)	Treated wastewater n=34 (%)	Surface water n=66 (%)	Drinking water n=11 (%)	Groundwater n=12 (%)	Others n=21 (%)
Jordan	1 (0.5)						1 (4.8)
Morocco	1 (0.5)						1 (4.8)
New Caledonia (France)	1 (0.5)	1 (1.8)					
Philippines	1 (0.5)			1 (1.5)			
Saudi Arabia	1 (0.5)	1 (1.8)					
Serbia	1 (0.5)			1 (1.5)			
Slovenia	1 (0.5)		1 (2.9)				
**WHO Region**							
Europe	74 (37.0)	27 (48.2)	16 (47.1)	21 (31.8)	3 (27.3)	2 (16.7)	5 (23.8)
America	49 (24.5)	10 (17.9)	5 (14.7)	24 (36.4)	1 (9.1)	6 (50.0)	3 (14.3)
Eastern Mediterranean	24 (12.0)	7 (12.5)	4 (11.8)	3 (4.6)	2 (18.2)		8 (38.1)
Africa	22 (11.0)	4 (7.1)	6 (17.7)	8 (12.1)	1 (9.1)	1 (8.3)	2 (9.5)
South-East Asia	15 (7.5)	5 (8.9)	2 (5.9)	4 (6.1)	3 (27.3)	1 (8.3)	
Western Pacific	15 (7.5)	2 (3.6)	1 (2.9)	6 (9.1)	1 (9.1)	2 (16.7)	3 (14.3)
Unclear	1 (0.5)	1 (1.8)					
**UNSD Region**							
Southern Europe	53 (26.5)	19 (33.9)	10 (29.4)	16 (24.2)	3 (27.3)	2 (16.7)	3 (14.3)
South America	26 (13.0)	6 (10.7)	4 (11.8)	14 (21.2)	1 (9.1)		1 (4.8)
Northern America	18 (9.0)	2 (3.6)		8 (12.1)		6 (50.0)	2 (9.5)
Southern Asia	16 (8.0)	5 (8.9)	2 (5.9)	3 (4.6)	3 (27.3)	1 (8.3)	2 (9.5)
Northern Africa	14 (7.0)	5 (8.9)	4 (11.8)	1 (1.5)			4 (19.1)
Southern Africa	13 (6.5)	1 (1.8)	5 (14.7)	5 (7.6)	1 (9.1)		1 (4.8)
Western Europe	13 (6.5)	4 (7.1)	4 (11.8)	4 (6.1)			1 (4.8)
Eastern Asia	11 (5.5)	1 (1.8)		4 (6.1)	1 (9.1)	2 (16.7)	3 (14.3)
Southeastern Asia	10 (5.0)	2 (3.6)	1 (2.9)	5 (7.6)	2 (18.2)		
Eastern Africa	8 (4.0)	3 (5.4)	1 (2.9)	3 (4.6)			1 (4.8)
Northern Europe	6 (3.0)	2 (3.6)	2 (5.9)	1 (1.5)			1 (4.8)

Characteristics	Overall n=200 (%)	Untreated wastewater n=56 (%)	Treated wastewater n=34 (%)	Surface water n=66 (%)	Drinking water n=11 (%)	Groundwater n=12 (%)	Others n=21 (%)
Central America	5 (2.5)	2 (3.6)	1 (2.9)	2 (3.0)			
Western Asia	5 (2.5)	3 (5.4)					2 (9.5)
Central Africa	1 (0.5)					1 (8.3)	
Oceania	1 (0.5)	1 (1.8)					
**Country income level**							
High-income economies	107 (53.5)	33 (58.9)	17 (50.0)	33 (50.0)	3 (27.3)	10 (83.3)	11 (52.4)
Upper-middle-income economies	54 (27.0)	10 (17.9)	10 (29.4)	25 (37.9)	5 (45.5)		4 (19.1)
Lower-middle income economies	33 (16.5)	12 (21.4)	6 (17.7)	6 (9.1)	3 (27.3)	1 (8.3)	5 (23.8)
Low-income economies	6 (3.0)	1 (1.8)	1 (2.9)	2 (3.0)		1 (8.3)	1 (4.8)
**HAV_detection_method**							
Conventional RT-PCR	109 (54.5)	34 (60.7)	16 (47.1)	32 (48.5)	6 (54.6)	9 (75.0)	12 (57.1)
Real-time RT-PCR	75 (37.5)	16 (28.6)	16 (47.1)	30 (45.5)	3 (27.3)	2 (16.7)	8 (38.1)
ICC Conventional RT-PCR	5 (2.5)	1 (1.8)		2 (3.0)	1 (9.1)		1 (4.8)
Immunocapture RT-PCR	5 (2.5)	2 (3.6)	1 (2.9)	1 (1.5)		1 (8.3)	
Molecular hybridization	3 (1.5)	2 (3.6)	1 (2.9)				
Countercurrent immuno-osmophoresis	1 (0.5)				1 (9.1)		
Culture + Electron microscopy	1 (0.5)	1 (1.8)					
Direct ELISA	1 (0.5)			1 (1.5)			
**Process control**							
Yes	74 (37.0)	15 (26.8)	11 (32.4)	33 (50.0)	2 (18.2)	5 (41.7)	8 (38.1)
Unclear/ Not reported	126 (63.0)	41 (73.2)	23 (67.7)	33 (50.0)	9 (81.8)	7 (58.3)	13 (61.9)
**Risk of bias**							

Characteristics	**Overall** **n=200** (%)	**Untreated wastewater** **n=56** (%)	**Treated wastewater** **n=34** (%)	**Surface water** **n=66** (%)	**Drinking water** **n=11** (%)	**Groundwater n=12** (%)	**Others** **n=21** (%)
Moderate risk of bias	187 (93.5)	51 (91.1)	31 (91.2)	63 (95.5)	11 (100)	10 (83.3)	21 (100)
Low risk of bias	13 (6.5)	5 (8.9)	3 (8.8)	3 (4.6)		2 (16.7)	

Table S7: Detailed results of meta-analysis results for prevalence of hepatitis A virus in various water matrices.

	**Prevalence (%) [95%CI]**	**95% Prediction interval**	**Number of studies**	**Number of samples**	**H [95%CI]**	**I^2 [95%CI]**	**P hetero-geneity**	**P difference subtypes**
**HAV prevalence in water matrices**								
**Countries**								< 0.001
Kenya	52 [0-100]	[0-100]	3	101	7.3 [5.4-9.8]	98.1 [96.5-99]	< 0.001	
Tunisia	39.1 [21.8-57.8]	[0-96.6]	9	897	5.5 [4.6-6.6]	96.7 [95.2-97.7]	< 0.001	
Uganda	36.8 [20.7-54.3]	[0-89.7]	5	97	1.7 [1-2.7]	63.9 [4.8-86.3]	0.026	
Singapore	35.4 [2.4-79.1]	[0-100]	3	81	3.8 [2.5-6]	93.2 [83.4-97.2]	< 0.001	
Sweden	33.3 [0-83.3]	[0-100]	3	49	3.4 [2.2-5.5]	91.6 [78.4-96.7]	< 0.001	
Brazil	32.5 [16.5-50.9]	[0-100]	22	1121	6.1 [5.5-6.8]	97.3 [96.7-97.8]	< 0.001	
Egypt	31.8 [8.4-61.3]	[0-100]	4	143	3.6 [2.4-5.2]	92.2 [83.2-96.4]	< 0.001	
South Africa	28.5 [13-47]	[0-95.6]	13	1235	6.2 [5.4-7.1]	97.4 [96.5-98]	< 0.001	
Spain	25.5 [12.7-40.6]	[0-86.2]	14	1146	4.7 [4-5.5]	95.5 [93.8-96.7]	< 0.001	
France	19.5 [4.1-41.3]	[0-97.3]	11	520	5 [4.2-6]	96 [94.3-97.2]	< 0.001	
India	14.3 [1.7-34.4]	[0-91.4]	9	911	6.5 [5.5-7.6]	97.6 [96.7-98.3]	< 0.001	
Argentina	12.9 [6.6-20.6]	[0-92.8]	3	169	1.2 [1-3.7]	29.2 [0-92.6]	0.244	
Italy	11.5 [5.9-18.4]	[0-56.1]	29	1857	3.6 [3.2-4.1]	92.4 [90.2-94.1]	< 0.001	
United States of America	8 [3-14.6]	[0-43]	17	2202	3.9 [3.3-4.6]	93.4 [90.8-95.2]	< 0.001	
Costa Rica	7.3 [3.7-11.8]	[0-47.8]	3	176	1 [1-3.1]	0 [0-89.6]	0.741	
Pakistan	4.8 [0.7-11.3]	[0-29.8]	6	235	1.6 [1-2.5]	62.1 [7.6-84.4]	0.022	

	Prevalence (%) [95%CI]	95% Prediction interval	Number of studies	Number of samples	H [95%CI]	I^2 [95%CI]	P hetero-geneity	P difference subtypes
Thailand	3.1 [0-9.4]	[0-33.2]	6	380	2.3 [1.6-3.4]	81.7 [61-91.4]	< 0.001	
Greece	1 [0-4.7]	[0-17.5]	6	428	1.7 [1.1-2.7]	66.8 [21-86.1]	0.01	
South Korea	0.4 [0-1.9]	[0-6.3]	10	940	1.5 [1.1-2.2]	57.3 [13.6-78.9]	0.012	
United Kingdom	0 [0-1.4]	[0-31.5]	3	143	1 [1-3.1]	0 [0-89.6]	0.994	
**WHO Region**								0.001
Africa	31.8 [16.9-48.8]	[0-99.5]	22	1445	6 [5.4-6.7]	97.3 [96.6-97.8]	< 0.001	
Eastern Mediterranean	23.7 [13.4-35.6]	[0-85.5]	24	1456	4.8 [4.3-5.5]	95.7 [94.6-96.6]	< 0.001	
America	20 [13-27.9]	[0-80.7]	49	5060	6.2 [5.7-6.6]	97.4 [97-97.7]	< 0.001	
Europe	12.9 [8.8-17.7]	[0-61.4]	74	5113	4.3 [4-4.6]	94.6 [93.7-95.3]	< 0.001	
South-East Asia	8.8 [1.8-19.4]	[0-65.7]	15	1291	5.1 [4.4-5.9]	96.1 [94.8-97.1]	< 0.001	
Western Pacific	4.7 [0.8-10.5]	[0-36.4]	15	1045	3 [2.5-3.7]	89.2 [83.9-92.8]	< 0.001	
**UNSD Region**								< 0.001
Eastern Africa	43.2 [12.5-77]	[0-100]	8	198	4.7 [3.8-5.8]	95.5 [93-97.1]	< 0.001	
Northern Africa	34.4 [20.7-49.4]	[0-91.2]	14	1090	4.9 [4.2-5.7]	95.9 [94.4-97]	< 0.001	
South America	30 [17.4-44.2]	[0-96.5]	26	1578	5.7 [5.1-6.3]	96.9 [96.2-97.5]	< 0.001	
Southern Africa	28.5 [13-47]	[0-95.6]	13	1235	6.2 [5.4-7.1]	97.4 [96.5-98]	< 0.001	
Central America	18.8 [1.7-46.4]	[0-100]	5	248	4.7 [3.5-6.3]	95.5 [92-97.4]	< 0.001	
Western Europe	18.1 [5.5-35.1]	[0-88.5]	13	653	4.6 [3.9-5.4]	95.3 [93.4-96.6]	< 0.001	
Western Asia	18 [5.1-35.9]	[0-88.1]	5	523	4.2 [3.1-5.7]	94.3 [89.4-96.9]	< 0.001	
Southern Asia	13.1 [3.6-26.4]	[0-79.3]	16	1164	5.2 [4.6-6]	96.4 [95.2-97.3]	< 0.001	
Southeastern Asia	13 [3.6-26.4]	[0-69.7]	10	473	3.4 [2.7-4.3]	91.5 [86.6-94.7]	< 0.001	
Southern Europe	11.4 [7.3-16.3]	[0-53.8]	53	3858	3.9 [3.6-4.3]	93.4 [92.1-94.5]	< 0.001	
Northern Europe	9.4 [0-35.1]	[0-99.7]	6	192	4.1 [3.1-5.5]	94.2 [89.9-96.6]	< 0.001	
Northern America	6.7 [2.9-11.7]	[0-32.9]	18	3234	3.8 [3.2-4.4]	93 [90.4-94.9]	< 0.001	
Eastern Asia	0.3 [0-1.6]	[0-5.6]	11	952	1.5 [1-2]	52.6 [5.9-76.1]	0.021	

	Prevalence (%) [95%CI]	95% Prediction interval	Number of studies	Number of samples	H [95%CI]	I^2 [95%CI]	P hetero-geneity	P difference subtypes
**Country income level**								0.002
Low-income economies	29 [11.8-49.6]	[0-92.4]	6	109	2.1 [1.4-3.1]	77.6 [50.2-89.9]	< 0.001	
Lower-middle income economies	24.2 [13-37.3]	[0-96.1]	33	2349	6.7 [6.1-7.2]	97.7 [97.3-98.1]	< 0.001	
Upper-middle-income economies	22.9 [15.4-31.3]	[0-86.9]	54	3636	5.5 [5.2-6]	96.7 [96.2-97.2]	< 0.001	
High-income economies	10.8 [7.8-14.1]	[0-52.2]	107	9328	4.3 [4-4.5]	94.6 [93.9-95.2]	< 0.001	
**Study period range**								0.853
[1986-2000]	14.5 [7.5-23.1]	[0-65.7]	25	1774	4.3 [3.7-4.8]	94.5 [92.9-95.7]	< 0.001	
[2000-2010]	17.3 [11.1-24.4]	[0-82.3]	67	3556	5 [4.7-5.4]	96 [95.4-96.5]	< 0.001	
[2010-2020]	15.5 [10.1-21.7]	[0-77.5]	75	5792	5.7 [5.3-6]	96.9 [96.5-97.2]	< 0.001	
**Environmental matrices categories**								< 0.001
Untreated wastewater	31.5 [23.1-40.5]	[0-92.8]	56	2840	4.8 [4.4-5.2]	95.6 [94.9-96.2]	< 0.001	
Treated wastewater	18 [9.5-28.3]	[0-83.9]	34	1649	4.7 [4.3-5.2]	95.5 [94.5-96.3]	< 0.001	
Surface water	14.6 [9.9-20]	[0-66.8]	66	5851	5.1 [4.8-5.5]	96.2 [95.6-96.7]	< 0.001	
Others	8.5 [3.2-15.7]	[0-50.8]	21	1529	3.7 [3.2-4.3]	92.6 [90-94.5]	< 0.001	
Groundwater	2.4 [0.2-6.1]	[0-20.9]	12	2165	3.2 [2.5-3.9]	90 [84.6-93.6]	< 0.001	
Drinking water	0.4 [0-1.8]	[0-6.2]	11	1388	1.7 [1.2-2.3]	64.4 [32-81.3]	0.002	
**HAV_detection_method**								< 0.001
Molecular hybridization	55 [23.9-84.2]	[0-100]	3	70	2.4 [1.3-4.1]	82.1 [44.7-94.2]	0.004	
Immunocapture RT-PCR	48.4 [20.6-76.8]	[0-100]	5	61	2.2 [1.4-3.4]	79.3 [50.9-91.3]	0.001	
Real-time RT-PCR	20.4 [13.5-28.3]	[0-90.8]	75	6781	7.2 [6.8-7.6]	98.1 [97.9-98.3]	< 0.001	
Conventional RT-PCR	13.3 [10.2-16.8]	[0-55.5]	109	7535	3.8 [3.6-4.1]	93.2 [92.3-94]	< 0.001	
ICC Conventional RT-PCR	1.1 [0-11.1]	[0-64.9]	5	669	4.8 [3.6-6.3]	95.6 [92.3-97.5]	< 0.001	

Table S5: Individual characteristics of included studies (provided as excel file)

Table S6: Risk of bias assessment (provided as excel file)

Figure S1: Global prevalence estimate of hepatitis A virus in various water matrices (provided as pdf file)

Figure S2: Funnel chart for publications of the global hepatitis A virus prevalence in water matrices (provided as pdf file)
